# Development and Validation of a Machine Learning Model for Detection and Classification of Tertiary Lymphoid Structures in Gastrointestinal Cancers

**DOI:** 10.1001/jamanetworkopen.2022.52553

**Published:** 2023-01-24

**Authors:** Zhe Li, Yuming Jiang, Bailiang Li, Zhen Han, Jeanne Shen, Yong Xia, Ruijiang Li

**Affiliations:** 1 School of Computer Science and Engineering, Northwestern Polytechnical University, Xi’an, China; 2 Department of Radiation Oncology, Stanford University School of Medicine, Stanford, California; 3 Department of General Surgery, Nanfang Hospital, Southern Medical University, Guangzhou, Guangdong, China; 4 Department of Pathology, Stanford University School of Medicine, Stanford, California

## Abstract

**Importance:**

Tertiary lymphoid structures (TLSs) are associated with a favorable prognosis and improved response to cancer immunotherapy. The current approach for evaluation of TLSs is limited by interobserver variability and high complexity and cost of specialized imaging techniques.

**Objective:**

To develop a machine learning model for automated and quantitative evaluation of TLSs based on routine histopathology images.

**Design, Setting, and Participants:**

In this multicenter, international diagnostic/prognostic study, an interpretable machine learning model was developed and validated for automated detection, enumeration, and classification of TLSs in hematoxylin-eosin–stained images. A quantitative scoring system for TLSs was proposed, and its association with survival was investigated in patients with 1 of 6 types of gastrointestinal cancers. Data analysis was performed between June 2021 and March 2022.

**Main Outcomes and Measures:**

The diagnostic accuracy for classification of TLSs into 3 maturation states and the association of TLS score with survival were investigated.

**Results:**

A total of 1924 patients with gastrointestinal cancer from 7 independent cohorts (median [IQR] age ranging from 57 [49-64] years to 68 [58-77] years; proportion by sex ranging from 214 of 409 patients who were male [52.3%] to 134 of 155 patients who were male [86.5%]). The machine learning model achieved high accuracies for detecting and classifying TLSs into 3 states (TLS1: 97.7%; 95% CI, 96.4%-99.0%; TLS2: 96.3%; 95% CI, 94.6%-98.0%; TLS3: 95.7%; 95% CI, 93.9%-97.5%). TLSs were detected in 62 of 155 esophageal cancers (40.0%) and up to 267 of 353 gastric cancers (75.6%). Across 6 cancer types, patients were stratified into 3 risk groups (higher and lower TLS score and no TLS) and survival outcomes compared between groups: higher vs lower TLS score (hazard ratio [HR]; 0.27; 95% CI, 0.18-0.41; *P* < .001) and lower TLS score vs no TLSs (HR, 0.65; 95% CI, 0.56-0.76; *P* < .001). TLS score remained an independent prognostic factor associated with survival after adjusting for clinicopathologic variables and tumor-infiltrating lymphocytes (eg, for colon cancer: HR, 0.11; 95% CI, 0.02-0.47; *P* = .003).

**Conclusions and Relevance:**

In this study, an interpretable machine learning model was developed that may allow automated and accurate detection of TLSs on routine tissue slide. This model is complementary to the cancer staging system for risk stratification in gastrointestinal cancers.

## Key Points

**Question:**

Can machine learning automatically evaluate tertiary lymphoid structures (TLSs) in histopathology images, and are quantitative scores of TLSs associated with survival?

**Findings:**

In this diagnostic/prognostic study of 1924 patients with gastrointestinal cancers, an interpretable machine learning model achieved accuracies greater than 95% for detecting and classifying TLSs into 3 maturation states in hematoxylin-eosin–stained images. The quantitative TLS score was an independent prognostic factor associated with survival after adjusting for clinicopathologic variables across 6 types of gastrointestinal cancers.

**Meaning:**

These findings suggest that a machine learning model may allow automated, accurate evaluation of TLSs on routine tissue slides, which is complementary to the cancer staging system for risk stratification.

## Teaser

This diagnostic/prognostic study investigates the use of a machine learning model in evaluating tertiary lymphoid structures and their association with survival in gastrointestinal cancers.

## Introduction

Tertiary lymphoid structures (TLSs) are ectopic lymphoid organs that develop in nonlymphoid tissues, such as sites of chronic inflammation and tumors.^1,2^ While the biological mechanisms behind their formation are incompletely understood, TLSs are known to play an important role in antitumor immune response.^3^ Indeed, the presence of TLSs has been associated with a favorable prognosis and improved response to immunotherapy across many cancer types.^4,5,6,7^

Currently, the most common and well-accepted approach to TLS detection is tissue staining for markers of immune cell lineages by multiplex immunohistochemistry or immunofluorescence techniques.^8,9,10^ However, multiplex imaging is not routinely applicable given its cost, high complexity, small field of view, and difficulty to scale, which limit its use to research settings. On the other hand, hematoxylin-eosin (H&E) staining is widely available and remains the clinical standard in histopathology. Therefore, detection of a TLSs on an H&E-stained tissue slide may provide a practical alternative to the current approach based on multiplex imaging.

Several groups have evaluated TLSs in routine H&E-stained slides based on pathologist assessment.^11^ However, this approach is time and labor intensive; manual and qualitative evaluation is further limited by interobserver variability.^12^ There is an unmet need for validated methods that allow standardized and quantitative evaluation of TLSs in H&E images.

Machine learning techniques are increasingly used to extract clinically relevant information from digital pathology data.^13,14,15^ Numerous studies have demonstrated the feasibility of deep learning for automated cancer diagnosis, grading, prediction of genetic alterations, and prognosis from H&E images.^16,17,18,19,20,21,22,23,24,25,26,27,28^ In this study, we aimed to develop an interpretable machine learning model for automated detection, enumeration, and classification of TLSs in whole-slide H&E images. Furthermore, we assessed the prognostic value of TLSs across multiple gastrointestinal cancers.

## Methods

This diagnostic/prognostic study followed the Transparent Reporting of a Multivariable Prediction Model for Individual Prognosis or Diagnosis (TRIPOD) reporting guideline. The use of public data sets from The Cancer Genome Atlas (TCGA) was determined by Stanford University to be exempt from review by institutional review boards (IRBs) in accordance with 45 CFR §46 because the study involved the use of publicly available data. Ethical approval for use of the Southern Medical University (SMU) data set was obtained from the IRB of SMU, and patient informed consent was waived by SMU for this retrospective analysis because the research could not practicably be carried out without using the information or biospecimens in an identifiable form.

### Study Design

We proposed a machine learning–based computational imaging analysis pipeline for fully automated and quantitative evaluation (including enumeration and characterization) of TLSs in routine H&E-stained whole-slide images. We further evaluated the prognostic significance of TLSs in 6 common cancers of the human digestive system: esophageal, gastric, colon, rectal, liver, and pancreatic cancer.

The overall study design and workflow is outlined in eFigure 1 in Supplement 1. Briefly, we first used a convolutional neural network with deep residual learning (ResNet18) to segment tumor vs normal tissue in whole-slide images. Next, we performed single-cell imaging analysis by a mask region–based convolutional neural network (R-CNN) to segment and classify individual nuclei into 3 cell types: lymphocytes, tumor cells, and other nonmalignant cells. Given the lymphocyte density map, we performed image processing and trained a machine learning model to obtain segmentation and classification for TLSs. Finally, we computed quantitative TLS scores for each tumor, which were then associated with patient prognosis and correlated with gene expression profiles. For a detailed description of image preprocessing, training of ResNet18 and Mask RCNN, and correlative analysis with molecular features, please refer to the eMethods in Supplement 1.

### Patients and Data Sets

In this international multicenter study, we retrospectively collected and analyzed whole-slide H&E images and clinical data for 1924 patients. We included 7 independent cohorts: TCGA esophageal carcinoma (TCGA-ESCA), stomach adenocarcinoma (TCGA-STAD), colon adenocarcinoma (TCGA-COAD), rectum adenocarcinoma (TCGA-READ), liver hepatocellular carcinoma (TCGA-LIHC), and pancreatic adenocarcinoma (TCGA-PAAD) and SMU-STAD. Digitized H&E whole-slide images are publicly available for TCGA cohorts. A total of 1813 whole-slide images were retrieved from The Cancer Imaging Archive, and 1660 images for 1592 patients were analyzed for TCGA cohorts. Most patients in TCGA had 1 slide; for patients with multiple slides, all available slides were analyzed. Additionally, 332 digitized H&E whole-slide images for 332 patients with gastric cancer were collected from the Nanfang Hospital of SMU in Guangzhou, China. All samples were obtained from resection specimens of treatment-naïve primary tumors. Only diagnostic slides generated from formalin-fixed, paraffin-embedded tumor sections were included. Detailed inclusion and exclusion criteria are described in eFigure 2 in Supplement 1.

### Segmentation and Classification of TLSs

We developed a computational pipeline to automatically identify, segment, and classify individual TLSs. First, based on the nuclei segmentation and lymphocyte mask obtained previously, we computed the number of lymphocytes per unit square on a 16 × 16 μm^2^ grid, producing lymphocyte density maps. Then, we applied thresholding and morphological image processing (opening operation) to these density maps, and after excluding lymphocyte clusters that were too small (ie, area <0.0384 mm^2^), we obtained final TLS segmentation masks.

In this study, 3 grades of TLSs were defined according to the degree of maturation: lymphoid aggregates, primary follicles, and secondary follicles with a germinal center. We trained a machine learning model to classify individual TLSs by grade. To do this, we selected 45 patients in the TCGA-STAD cohort and identified a total of 865 TLSs, which were manually labeled as 1 of 3 TLS grades by a pathologist (Y.J.). The whole data set was randomly partitioned into a training set (379 TLSs) and testing set (486 TLSs). Given that TLS2 and TLS3 tend to have a round shape and are usually larger than TLS1 and that TLS3 has a unique germinal center with lower lymphocyte density, we computed 3 features for each TLS: area, roundness (ie, ratio of the area multiplied by 4π to the square of the perimeter), and skewness of lymphocyte density for each TLS. Using these data, we trained a model using the classification and regression trees (CART) algorithm. We trained CART with the scikit-learn package from the Python programming language version 3.6.11 (Python Software Foundation) using default parameter settings (criterion = gini; splitter = best; min_samples_split = 2). The maximum depth of trees was determined to be 4 using 5-fold cross validation in the training set. Given the relative importance of TLS3, class weights for TLS1, TLS2, and TLS3 were empirically set to 1, 2, and 3, respectively.

### Quantitative Scoring of TLSs

We first calculated the total TLS area for each grade (TLS1, TLS2, and TLS3) and tumor area. For each patient, 3 individual TLS scores were computed as the total TLS area for each grade normalized by tumor area. We then defined the overall TLS score as the linear weighted sum of TLS area divided by tumor area as follows:

TLS score = (*w1* × area_TLS1_ + *w2* × area_TLS2_ + *w3* × area_TLS3_)/area_tumor_

where area_TLS1_, area_TLS2, _area_TLS3_, and area_tumor_ are the total TLS1, TLS2, TLS3, and tumor area, respectively, and *w*1, *w*2, and *w*3 are corresponding weights. To determine optimal weights, we performed Cox regression analysis of overall survival with TLS1 score, TLS2 score, and TLS3 score in the TCGA-STAD cohort.

### Statistical Analysis

We used overall accuracy and a confusion matrix to evaluate the performance for TLS classification. The prognostic value of individual and overall TLS scores were assessed by the association with survival outcomes. Overall survival was defined as the time from diagnosis to death or the last follow-up. Progression-free survival was defined as the time from diagnosis to disease progression, death, or the last follow-up. Univariate and multivariate analyses were performed with the Cox proportional hazard model. Clinical and pathological variables, such as tumor stage and grade, were included in the multivariate analysis. Kaplan-Meier analysis and the log-rank test were used to evaluate patient stratification by risk group. We used Harrel concordance index (*C* index) as a metric for assessing the performance of prognosis prediction. A 2-sided *P* value less than .05 was considered statistically significant. Data analysis was performed between June 2021 and March 2022 using the lifelines package version 0.25.11 in the Python programming langauge.

## Results

### Patient Characteristics

A total of 1924 patients with gastrointestinal cancer across 7 cohorts (median [IQR] age ranging from 57 [49-64] years for SMU-STAD to 68 [58-77] years for TCGA-COAD; proportion by sex ranging from 214 of 409 patients who were male [52.3%] for TCGA-COAD to 134 of 155 patients who were male [86.5%] for TCGA-ESCA) were included in the study. In most cancer types, AJCC stage II and III diseases accounted for most diagnoses (ranging from 164 of 355 cancers [45.2%] for TCGA-LIHC to 148 of 175 cancers [84.6%] for TCGA-PAAD). Gastric cancer data sets for TCGA and SMU had similar distributions of clinicopathologic features. Baseline characteristics for patients in the 7 cohorts are summarized in the Table.

**Table.** Clinicopathologic Characteristics of Patients by Cohort

Characteristic^a^	Patients, No. (%) (N = 1924)						
	TCGA-ESCA (n = 155)	TCGA-STAD (n = 353)	SMU-STAD (n = 332)	TCGA-COAD (n = 409)	TCGA-READ (n = 145)	TCGA-LIHC (n = 355)	TCGA-PAAD (n = 175)
Sex							
Female	21 (13.5)	122 (34.6)	101 (30.4)	195 (47.7)	63 (43.4)	116 (32.7)	77 (44.0)
Male	134 (86.5)	231 (65.4)	231 (69.6)	214 (52.3)	82 (56.6)	239 (67.3)	98 (56.0)
Age, median (IQR), y	59 (53-70)	66 (57-72)	57 (49-64)	68 (58-77)	65 (57-72)	61 (51-69)	65 (57-73)
Cancer stage							
I	15 (9.7)	41 (11.6)	44 (13.3)	71 (17.4)	27 (18.6)	164 (46.2)	20 (11.4)
II	66 (42.6)	111 (31.4)	73 (22.0)	150 (36.7)	42 (29.0)	83 (23.4)	145 (82.9)
III	48 (31.0)	161 (45.6)	175 (52.7)	121 (29.6)	46 (31.7)	81 (22.8)	3 (1.7)
IV	7 (4.5)	33 (9.3)	40 (12.0)	56 (13.7)	22 (15.2)	4 (1.1)	5 (2.9)
Unknown	19 (12.3)	7 (2.0)	0	11 (2.7)	8 (5.5)	23 (6.5)	2 (1.1)
Tumor grade							
1	18 (11.6)	7 (2.0)	41 (12.3)	0	0	48 (13.5)	30 (17.1)
2	76 (49.0)	123 (34.8)	70 (21.1)	0	0	170 (47.9)	91 (52.0)
3	44 (28.4)	214 (60.6)	221 (66.6)	0	0	119 (33.5)	49 (28.0)
4	0	0	0	0	0	13 (3.7)	2 (1.1)
Unknown	17 (11.0)	9 (2.5)	0	409 (100)	145 (100)	5 (1.4)	3 (1.7)

Abbreviations: COAD, colon adenocarcinoma; ESCA, esophageal carcinoma; LIHC, liver hepatocellular carcinoma; PAAD, pancreatic adenocarcinoma; READ, rectum adenocarcinoma; SMU, Southern Medical University; STAD, stomach adenocarcinoma; TCGA, The Cancer Genome Atlas.

### Accurate Tumor Detection and Classification of Tumor-Infiltrating Lymphocytes

The ResNet18 model for tumor detection achieved an out-of-sample area under the curve greater than 0.99 (95% CI, 0.98-1.00). We manually segmented 15368 nuclei in 140 image patches from 20 randomly selected patients with gastric cancer in the TCGA-STAD data set and labeled each nucleus as tumor cell, lymphocyte, or other cell. The Mask R-CNN model achieved an accuracy for nuclei detection of 91.1% (95% CI, 90.6%-91.5%), with precision and recall rates of 97.1% (95% CI, 96.8%-97.4%) and 93.7% (95% CI, 93.3%-94.1%), respectively. For nuclei classification, high accuracies were also observed, with 95.8% (95% CI, 95.5%-96.1%) in training and 95.37% (95% CI, 95.0%-95.7%) in testing data sets for tumor cells and 98.4% (95% CI, 98.2%-98.6%) in training and 95.7% (95% CI, 95.4%-96.0%) in testing data sets for lymphocytes (eFigure 3 in Supplement 1).

### Accurate Classification of TLS

**Figure 1.**
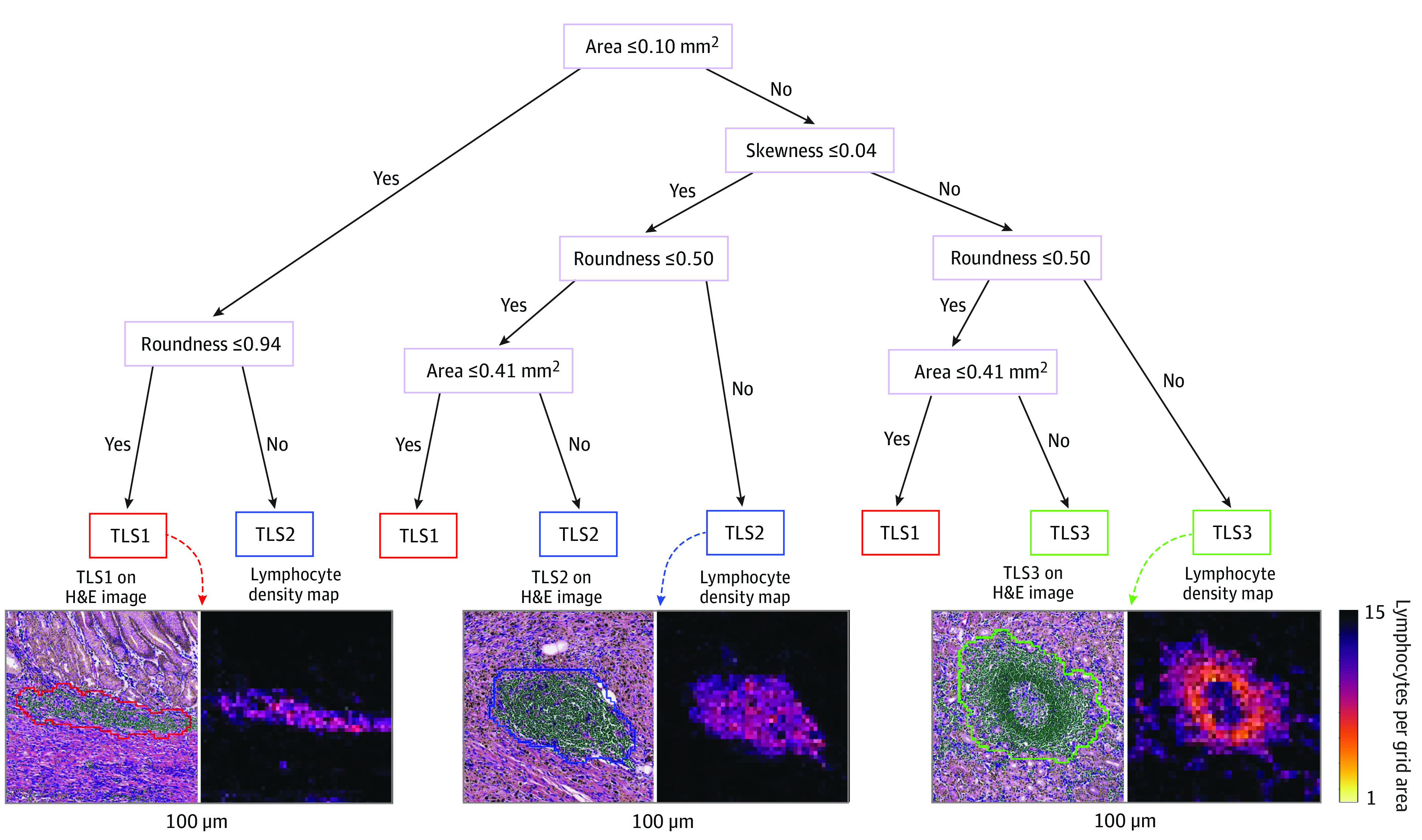
Automated Classification of Tertiary Lymphoid Structures (TLSs) A simple and interpretable decision tree model was used to classify TLSs into 1 of 3 grades based on 3 image features of TLSs: area, roundness, and skewness. A representative image patch containing TLSs and the corresponding lymphocyte density map is shown for each grade. H&E indicates hematoxylin-eosin.

**Figure 2.**
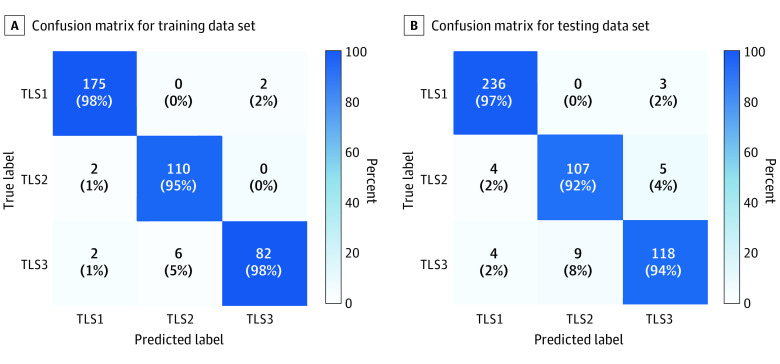
Confusion Matrices for Tertiary Lymphoid Structures (TLS) Classification Values are the percentage and number of TLSs correctly (diagonal) and incorrectly (off diagonal) classified by the decision tree.

The optimal decision tree to classify each TLS as TLS1, TLS2, or TLS3 is shown in Figure 1. The proposed model was accurate in the training data set, with an overall classification of 373 of 379 labels (98.4%; 95% CI, 97.1%-99.7%) for TLS1, 371 of 379 labels (97.9%; 95% CI, 96.5%-99.3%) for TLS2, and 369 of 379 labels (97.4%; 95% CI, 95.8%-99.0%) for TLS3. Similarly, high TLS classification accuracies were observed in the testing data set (TLS1: 97.7%; 95% CI, 96.4%-99.0%; TLS2: 96.3%; 95% CI, 94.6%-98.0%; TLS3: 95.7%; 95% CI, 93.9%-97.5%). Figure 2A and B shows confusion matrices for TLS classification in training and testing data sets. Some additional images for TLS classification are shown in eFigure 4 in Supplement 1.

### Whole-Slide Image–Based Enumeration and Quantitative Evaluation of TLSs

The percentage of tumors with at least 1 TLS detected is shown in eFigure 5 in Supplement 1. In all 6 cancer types, TLS1 was more frequently found than other types of TLS, and as high as 69% of tumors in TCGA-STAD had at least 1 TLS1 (242 of 353 tumors [68.6%]). The proportion of tumors with any type of TLS detected ranged from 62 of 155 tumors (40.0%) for TCGA-ESCA to 267 of 353 tumors (75.6%) for TCGA-STAD, indicating that TLSs were highly prevalent in gastrointestinal cancers (eFigure 5 in Supplement 1).

**Figure 3.**
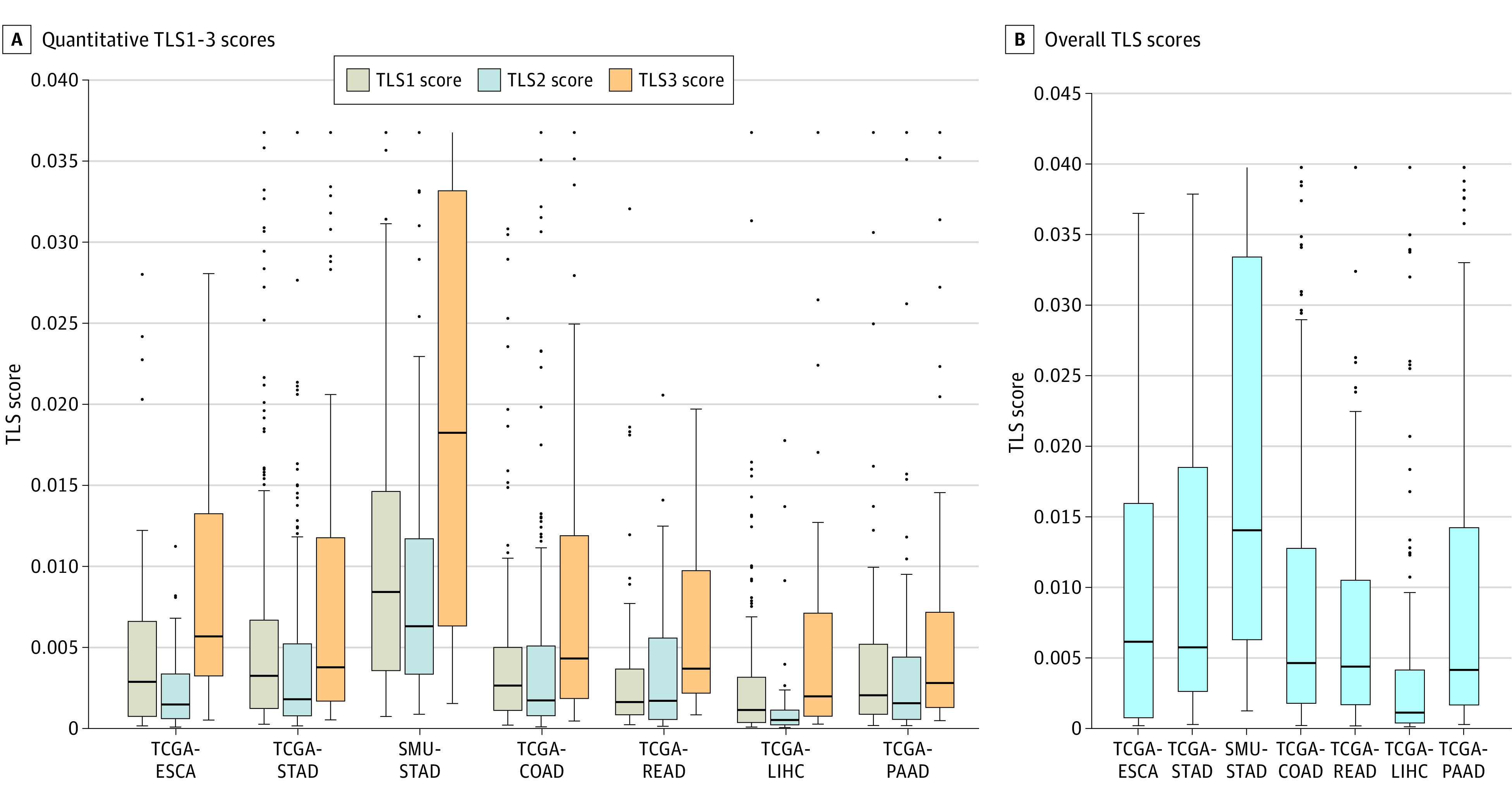
Distributions of Tertiary Lymphoid Structure (TLS) Scores The distributions of quantitative TLS1 through TLS3 scores and overall TLS score across 6 cancer types in 7 cohorts are shown. Boxes indicate first and third quartiles; COAD, colon adenocarcinoma; dots, outliers; ESCA, esophageal carcinoma; horizontal lines, medians; LIHC, liver hepatocellular carcinoma; PAAD, pancreatic adenocarcinoma; READ, rectum adenocarcinoma; SMU, Southern Medical University; STAD, stomach adenocarcinoma; TCGA, The Cancer Genome Atlas; whiskers, 1.5 × IQR.

The distributions of TLS scores by grade are shown in Figure 3A. We also computed TLS density, or the number of TLSs per unit tumor area, which had similar distributions to those of TLS scores (eFigure 6 in Supplement 1). Among 6 cancer types, the SMU-STAD cohort had the highest quantitative score and TLS density, and TCGA-READ had the lowest score for TLS. The mean (SD) TLS size for all patients was 0.009 (0.023) of the tumor area, while the mean (SD) TLS size for patients with any type of TLS detected was 0.016 (0.029) of the tumor area (Figure 3A). In rare cases, TLS size was 5% to 10% of the tumor area. Pearson correlation values were minor to modest among 3 individual TLS scores, suggesting that these scores may be complementary (eFigure 7 in Supplement 1).

To summarize TLS scores for each patient, we linearly combined 3 individual TLS scores. The optimal corresponding weights were 0.81 for TLS1, 0.84 for TLS2, and 1.00 for TLS3. The distributions of overall TLS scores across 7 cohorts are shown in Figure 3B. Similar to individual TLS scores, gastric cancer had the highest overall score for TLS; by contrast, liver cancer had the lowest TLS scores; while esophageal, colon, rectal, and pancreatic cancers had intermediate TLS scores. We then assessed the association between TLS scores and tumor stage or grade; we did not observe an association except for TCGA-STAD and TCGA-READ cancer, with higher TLS scores for stage II and III disease (eTable 1 in Supplement 1). We also evaluated the interslide variability of TLS scores in patients who had more than 1 slide available. The mean (SD) coefficient of variation (ie, ratio of the SD to mean TLS scores) ranged from 0.30 (0.35) in TCGA-LIHC to 0.57 (0.39) in TCGA-STAD, indicating small to moderate interslide variability (eTable 2 in Supplement 1).

### Prognostic Outcome of TLS Scores Across Cancer Types

**Figure 4.**
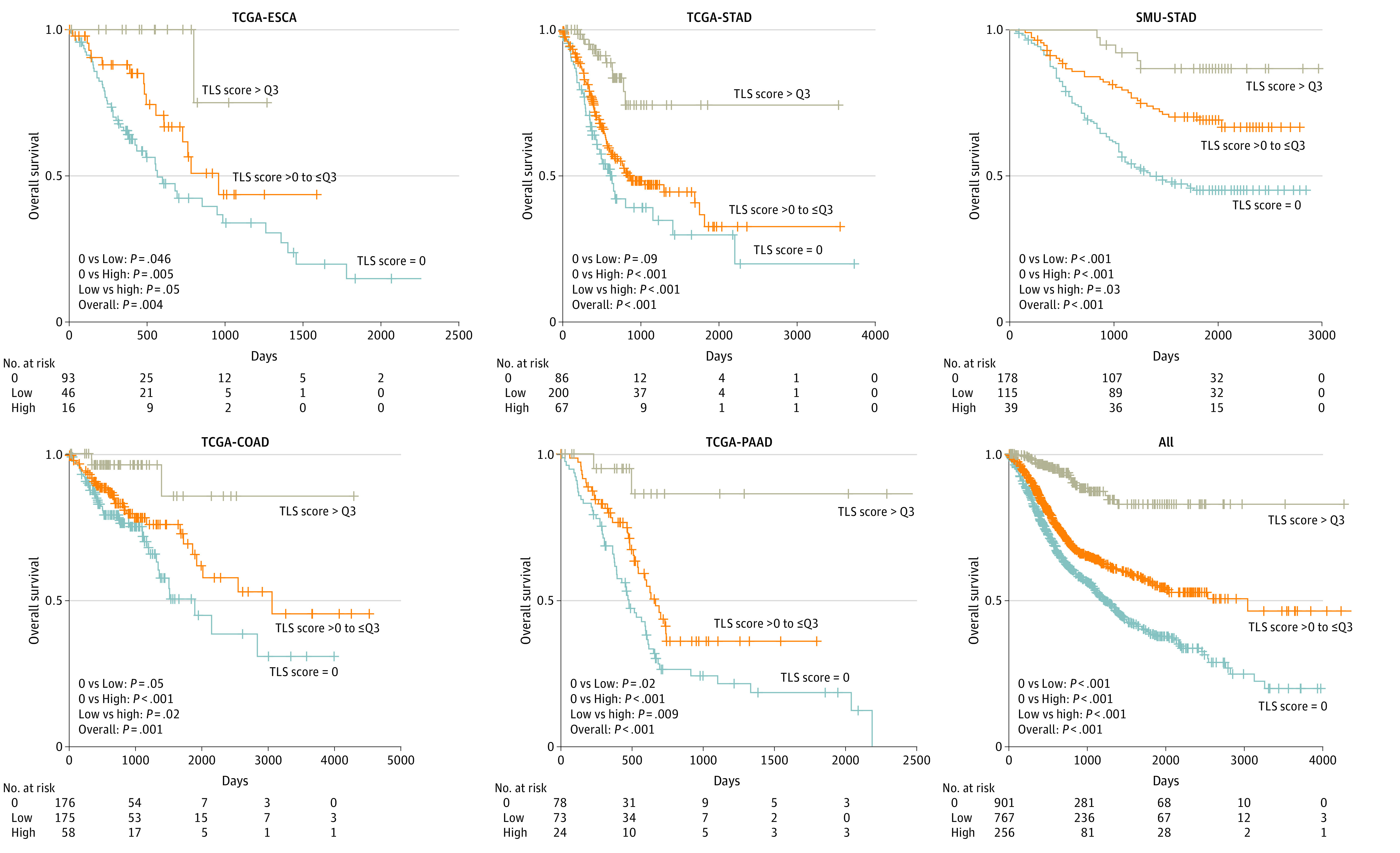
Prognostic Outcomes of Tertiary Lymphoid Structures (TLSs) Kaplan-Meier curves of overall survival for patients with high vs low overall TLS scores vs no TLSs are presented. *P* values were determined by 2-sided log-rank test. COAD indicates colon adenocarcinoma; ESCA, esophageal carcinoma; PAAD, pancreatic adenocarcinoma; Q3, upper quartile; SMU, Southern Medical University; STAD, stomach adenocarcinoma; TCGA, The Cancer Genome Atlas.

We assessed the prognostic outcome of TLS scores across 6 cancer types and 7 cohorts. The overall TLS score stratified patients into 3 distinct risk groups (Figure 4; eFigure 8 in Supplement 1). Patients with a higher TLS score had a significantly improved overall survival compared with patients with a lower TLS score (overall hazard ratio [HR], 0.27; 95% CI, 0.18-0.41; *P* < .001); these patients in turn had better survival than those with no TLSs detected (HR, 0.65; 95% CI, 0.56-0.76; *P* < .001). Comparing patients who had higher TLS scores with those with no TLSs, the difference in survival was larger (HR, 0.18; 95% CI, 0.12-0.27; *P* < .001).

The prognostic outcome of overall TLS scores showed the same pattern for progression-free survival (eFigure 9 in Supplement 1). Patients with a higher TLS score had a significantly improved progression-free survival compared with patients with a lower TLS score (HR, 0.56; 95% CI, 0.43-0.72; *P* < .001), who had better progression-free survival than those with no TLSs detected (HR, 0.72; 95% CI, 0.63-0.83; *P* < .001); patients with higher TLS scores also had better progression-free survival than those with no TLSs (HR, 0.41; 95% CI, 0.32-0.52; *P* < .001). Similar results were obtained for each of 3 individual TLS scores in all 7 cohorts (eFigure 10 in Supplement 1). In univariable analysis, each of 3 TLS scores was associated with overall survival in all cancers except TSL2 in TCGA-ESCA, COAD, and PAAD, as well as in rectal cancer, which had the smallest number of samples (eTable 3 in Supplement 1). For example, for TCGA-STAD, the HR was 0.55 (95% CI, 0.41-0.75; *P* = .001) for TLS1, 0.53 (95% CI, 0.34-0.82; *P* = .005) for TLS2, and 0.18 (95% CI, 0.07-0.51; *P* = .001) for TLS3. All 3 scores remained significant in multivariable analysis in the combined data set, with TLS3 being the strongest predictor of survival (for all data sets combined: HR, 0.25; 95% CI, 0.15-0.42; *P* < .001). Compared with individual TLS scores, the overall TLS score achieved higher accuracies for survival prediction (eFigure 11 in Supplement 1). Importantly, TLS scores showed a superior prognostication performance compared with TLS density, suggesting that quantitative scoring may provide additional information than simple enumeration of TLSs (eFigure 12 in Supplement 1). To investigate different weighting of individual TLS scores, we retrained a linear model using SMU-STAD, TCGA-PAAD, or combined data sets. Overall TLS scores remained stable, with Pearson correlations of 0.93 or greater, and prognostic patterns were similar to those in the original results (0.93 for PAAD and 0.96 for SMU-STAD in eFigure 22 in Supplement 1).

The overall TLS score predicted overall survival with an accuracy similar to or higher than that of tumor stage (eFigure 13 in Supplement 1). For rectal and pancreatic cancer, TLS score outperformed stage for survival prediction. To further improve prognostication, we combined tumor stage with TLS score, with optimal weights of −0.51 and 1.36, respectively. The combined model had a significantly improved *C* index for survival prediction compared with tumor stage (eFigure 13 in Supplement 1). In multivariable analysis that included clinicopathologic variables and density of tumor-infiltrating lymphocytes, the overall TLS score remained an independent prognostic factor in all 7 cohorts (eg, for colon cancer: HR, 0.11; 95% CI, 0.02-0.47; *P* = .003) (eTables 4-10 in Supplement 1). Within each subgroup of patients as defined by age, sex, tumor stage, and grade, the overall TLS score was associated with overall survival (eFigures 14-20 in Supplement 1). For patients with the same disease stage, the overall TLS score further stratified patients in most cancer types (eFigure 21 in Supplement 1).

Finally, we investigated molecular features associated with imaging-based TLS scores and developed a gene expression signature consisting of 11 cytokines (eFigures 23-24 and eTable 11 in Supplement 1). Results further suggested the independent prognostic value of TLS score in 1858 patients with gastric and colorectal cancers (eFigures 25-27 in Supplement 1). For a detailed description of weighting of individual TLS scores, molecular correlates, and the gene signature of the TLS score, please refer to the eResults in Supplement 1.

## Discussion

In this diagnostic/prognostic study, we developed an interpretable machine learning model for automated detection, enumeration, and classification of TLSs based on routine H&E-stained whole-slide images. Additionally, we proposed a quantitative scoring system for TLSs and confirmed its independent prognostic value in an international multicenter cohort of 1924 patients across 6 common gastrointestinal cancers. Finally, we developed a gene signature of the imaging-based TLS score, which further confirmed its prognostic value.

To our knowledge, this is the first and largest study to develop an automated quantitative TLS scoring system based on routine H&E-stained images and validate its clinical relevance in all major types of gastrointestinal cancers. Recently, the presence of TLSs has been associated with a favorable prognosis and improved response to immunotherapy in multiple cancer types.^4,5,6,7^ In previous studies,^4,5,6,7^ TLSs were identified using multiplexed immunohistochemistry staining or immunofluorescence imaging, which are not routinely used. In this study, we developed a computational approach for TLS evaluation based on routine H&E slides, which may be broadly applicable in a clinical setting.

We found a favorable prognostic outcome for TLSs across 6 gastrointestinal cancer types. These findings are consistent with those of previous studies on gastrointestinal cancers^29,30^ and other cancers.^8,9,10,31^ Of note, TLS3 with a germinal center had the largest weights in our TLS scoring, suggesting that mature TLSs may play the most important role in antitumor immune response, as shown in previous studies.^4,5,6,7^ By contrast, the density of tumor-infiltrating lymphocytes, which may be associated with lymphocyte aggregates (eg, TLS1), did not have an independent prognostic association after adjusting for TLS score. Importantly, we found that TLS score was independent of established prognostic factors, including tumor stage and grade, suggesting that its use may be associated with further refinement in the current staging system and improved risk stratification.

An important advantage of our approach was the automated enumeration and quantitative characterization of TLS. Previous work relied on manual and qualitative assessment of TLSs by the pathologist, which has been found to be inaccurate and subject to interobserver variability when assessed on H&E-stained slides.^12^ In this study, we developed an automated computational pipeline for TLS scoring, which may allow for standardized and quantitative evaluation of TLSs.

While deep learning has shown promising performance in digital pathology,^13,14^ most studies have adopted the patch- or tile-based approach for image analysis. Because TLSs are highly variable in size, density, and morphology, there are significant challenges for the traditional patch-based approach. Here, we developed a deep learning–based single-cell analysis tool that may allow automated segmentation and classification of tumor-infiltrating lymphocytes on whole-slide images. By quantifying the spatial distribution of lymphocytes, we developed an accurate and interpretable model for classification of TLSs according to their maturation states.

We found that the mean (SD) TLS size was less than 1% of the tumor area, although in rare cases, this reached 5% to 10%. This suggests that image-based detection of TLSs may exhibit more variability in small biopsies that are typically available for patients with metastatic cancer. This shortcoming may be overcome with a molecular approach that relies on secreted proteins, such as relevant cytokines and chemokines. To that end, we developed an 11-gene signature for TLSs. Unlike previous signatures that included genes positively associated with TLSs, our signature contains not only genes (ie, *CXCL13*, *CXCL11*, *CXCL10*) with an established role in TLS formation and function, but also genes (ie, *TGFB2* and *VEGFB*) associated with an immunosuppressive tumor microenvironment that may inhibit the development of TLSs.^3^

### Limitations

This study has several limitations. As a retrospective study, it has a potential for selection bias, although we tried to include all eligible patients with diverse characteristics and geographic locations. In gastric cancer, despite some differences in the quantity of TLSs between TCGA-STAD and SMU-STAD cohorts, prognostic patterns were similar. In our study, TLS identification and classification was performed by a pathologist based on H&E images. Ideally, the criterion standard for TLS detection is based on immunohistochemistry. We could not assess the association of certain established prognostic factors, such as lymphovascular invasion or liver cirrhosis, with survival outcomes given that this information was not available in TCGA cohorts. Additionally, our study did not include patients who received immunotherapy, and we could not confirm the predictive value of TLS scores for response to immunotherapy.

## Conclusions

In this multicenter diagnostic/prognostic study of 1924 patients, we developed a machine learning–based computational tool for automated detection and quantitative evaluation of TLSs on routine H&E slides and confirmed the association of TLSs with survival in gastrointestinal cancers. The proposed TLS scoring system may complement the current staging system and be associated with refinements in risk stratification. Prospective validation studies may be warranted to confirm that our results are reproducible and generalizable across broader patient populations.

## Notes

Supplement 1.
eMethods.

eResults.

eReferences.
**eFigure 1.** Proposed Workflow for Automated Tertiary Lymphoid Structure Evaluation on Hematoxylin-Eosin–Stained Whole-Slide Images **eFigure 2.** Flow Chart of Patient Inclusion and Exclusion **eFigure 3.** Confusion Matrices for Nuclei Classification on Training and Testing Data Set **eFigure 4.** Example Images for Tertiary Lymphoid Structure Segmentation and Classification **eFigure 5.** Distributions of Tertiary Lymphoid Structure Across 6 Cancer Types in 7 Cohorts **eFigure 6.** Distributions of Tertiary Lymphoid Structure Density in 7 Cohorts **eFigure 7.** Correlation Among 3 Individual Tertiary Lymphoid Structure Scores in 7 Cohorts **eFigure 8.** Prognostic Outcome of Tertiary Lymphoid Structure Score Across 3 Cancer Types in 4 Cohorts **eFigure 9.** Kaplan-Meier Curves of Progression-Free Survival for Patients With High vs Low Overall Tertiary Lymphoid Structure (TLS) Scores vs No TLSs **eFigure 10.** Kaplan-Meier Survival Analysis of Overall Survival by Individual Tertiary Lymphoid Structure 1-3 Score **eFigure 11.** Concordance Index of Overall Survival Predictions Using Overall Tertiary Lymphoid Structure (TLS) Score and Individual TLS1-3 Scores **eFigure 12.** Concordance Index of Overall Survival Predictions Using Tertiary Lymphoid Structure Score and Density **eFigure 13.** Comparison of C Index for Predicting Overall Survival Using Tertiary Lymphoid Structure Score, Tumor Stage, and Combined Model **eFigure 14.** Forest Plot of Tertiary Lymphoid Structure Score for The Cancer Genome Atlas Esophageal Carcinoma **eFigure 15.** Forest Plot of Tertiary Lymphoid Structure Score for The Cancer Genome Atlas Stomach Adenocarcinoma **eFigure 16.** Forest Plot of Tertiary Lymphoid Structure Score for Southern Medical University Stomach Adenocarcinoma **eFigure 17.** Forest Plot of Tertiary Lymphoid Structure Score for The Cancer Genome Atlas Colon Adenocarcinoma **eFigure 18.** Forest Plot of Tertiary Lymphoid Structure Score for The Cancer Genome Atlas Rectum Adenocarcinoma **eFigure 19.** Forest Plot of Tertiary Lymphoid Structure Score for The Cancer Genome Atlas Liver Hepatocellular Carcinoma **eFigure 20.** Forest Plot of Tertiary Lymphoid Structure Score for The Cancer Genome Atlas Pancreatic Adenocarcinoma **eFigure 21.** Kaplan-Meier Survival Analysis by Tertiary Lymphoid Structure Score for Patients With Same Tumor Stage **eFigure 22.** Comparison of Tertiary Lymphoid Structure Scores Calculated Using Weights Trained on Different Cohorts **eFigure 23.** Correlation Between Tertiary Lymphoid Structure Scores and 10 Tumor Microenvironmental Cell Types Estimated From Gene Expression Data in The Cancer Genome Atlas Stomach Adenocarcinoma Cohort **eFigure 24.** Gene Expression Profile of 23 Cytokines Correlated With Tertiary Lymphoid Structure Score in The Cancer Genome Atlas Stomach Adenocarcinoma Cohort **eFigure 25.** Molecular Signature of Tertiary Lymphoid Structure Score and Prognostic Value **eFigure 26.** Multivariate Survival Analysis of Tertiary Lymphoid Structure Molecular Signature in Combined Gastric Cancer Gene Expression Data Sets **eFigure 27.** Multivariate Survival Analysis of Tertiary Lymphoid Structure Molecular Signature in Combined Colorectal Cancer Gene Expression Data Sets **eTable 1.** Association Between Tertiary Lymphoid Structure Score and Tumor Stage or Grade in 7 Cohorts **eTable 2.** Interslide Variability of Tertiary Lymphoid Structure Scores **eTable 3.** Univariate and Multivariate Survival Analysis of Individual Tertiary Lymphoid Structure Scores in 7 Cohorts and Combined Data Set **eTable 4.** Univariate and Multivariate Survival Analysis of Overall Tertiary Lymphoid Structure Scores in The Cancer Genome Atlas Esophageal Carcinoma **eTable 5.** Univariate and Multivariate Survival Analysis of Overall Tertiary Lymphoid Structure Scores in The Cancer Genome Atlas Stomach Adenocarcinoma **eTable 6.** Univariate and Multivariate Survival Analysis of Overall Tertiary Lymphoid Structure Scores in Southern Medical University Stomach Adenocarcinoma **eTable 7.** Univariate and Multivariate Survival Analysis of Overall Tertiary Lymphoid Structure Scores in The Cancer Genome Atlas Colon Adenocarcinoma **eTable 8.** Univariate and Multivariate Survival Analysis of Overall Tertiary Lymphoid Structure Scores in The Cancer Genome Atlas Rectum Adenocarcinoma **eTable 9.** Univariate and Multivariate Survival Analysis of Overall Tertiary Lymphoid Structure Scores in The Cancer Genome Atlas Liver Hepatocellular Carcinoma **eTable 10.** Univariate and Multivariate Survival Analysis of Overall Tertiary Lymphoid Structure Scores in The Cancer Genome Atlas Pancreatic Adenocarcinoma **eTable 11.** The 11 Cytokine Genes and Corresponding Weights in Tertiary Lymphoid Structure Molecular Signature

Supplement 2.
Data Sharing Statement
