# Supplementary material for: Development and Validation of a Machine Learning Model for Detection and Classification of Tertiary Lymphoid Structures in Gastrointestinal Cancers
Source: JAMA Netw Open. 2023 Jan 24;6(1):e2252553. doi: 10.1001/jamanetworkopen.2022.52553 (PMC10408275; doi:10.1001/jamanetworkopen.2022.52553)
Supplement: Supplement 1. — eMethods. eResults. eReferences. eFigure 1. Proposed Workflow for Automated Tertiary Lymphoid Structure Evaluation on Hematoxylin-Eosin–Stained Whole-Slide Images eFigure 2. Flow Chart of Patient Inclusion and Exclusion eFigure 3. Confusion Matrices for Nuclei Classification on Training and Testing Data Set eFigure 4. Example Images for Tertiary Lymphoid Structure Segmentation and Classification eFigure 5. Distributions of Tertiary Lymphoid Structure Across 6 Cancer Types in 7 Cohorts eFigure 6. Distributions of Tertiary Lymphoid Structure Density in 7 Cohorts eFigure 7. Correlation Among 3 Individual Tertiary Lymphoid Structure Scores in 7 Cohorts eFigure 8. Prognostic Outcome of Tertiary Lymphoid Structure Score Across 3 Cancer Types in 4 Cohorts eFigure 9. Kaplan-Meier Curves of Progression-Free Survival for Patients With High vs Low Overall Tertiary Lymphoid Structure (TLS) Scores vs No TLSs eFigure 10. Kaplan-Meier Survival Analysis of Overall Survival by Individual Tertiary Lymphoid Structure 1-3 Score eFigure 11. Concordance Index of Overall Survival Predictions Using Overall Tertiary Lymphoid Structure (TLS) Score and Individual TLS1-3 Scores eFigure 12. Concordance Index of Overall Survival Predictions Using Tertiary Lymphoid Structure Score and Density eFigure 13. Comparison of C Index for Predicting Overall Survival Using Tertiary Lymphoid Structure Score, Tumor Stage, and Combined Model eFigure 14. Forest Plot of Tertiary Lymphoid Structure Score for The Cancer Genome Atlas Esophageal Carcinoma eFigure 15. Forest Plot of Tertiary Lymphoid Structure Score for The Cancer Genome Atlas Stomach Adenocarcinoma eFigure 16. Forest Plot of Tertiary Lymphoid Structure Score for Southern Medical University Stomach Adenocarcinoma eFigure 17. Forest Plot of Tertiary Lymphoid Structure Score for The Cancer Genome Atlas Colon Adenocarcinoma eFigure 18. Forest Plot of Tertiary Lymphoid Structure Score for The Cancer Genome Atlas Rectum Adenocarcinoma eFigure 19. Forest [file jamanetwopen-e2252553-s001.pdf]

Supplemental Online Content

Li Z, Jiang Y, Li B, et al. Development and validation of a machine learning model for detection and classification of tertiary lymphoid structures in gastrointestinal cancers. *JAMA Netw Open*. 2023;6(1):e2252553. doi:10.1001/jamanetworkopen.2022.52553

eMethods.
eResults.
eReferences.
eFigure 1. Proposed Workflow for Automated Tertiary Lymphoid Structure Evaluation on Hematoxylin-Eosin–Stained Whole-Slide Images
eFigure 2. Flow Chart of Patient Inclusion and Exclusion
eFigure 3. Confusion Matrices for Nuclei Classification on Training and Testing Data Set
eFigure 4. Example Images for Tertiary Lymphoid Structure Segmentation and Classification
eFigure 5. Distributions of Tertiary Lymphoid Structure Across 6 Cancer Types in 7 Cohorts
eFigure 6. Distributions of Tertiary Lymphoid Structure Density in 7 Cohorts
eFigure 7. Correlation Among 3 Individual Tertiary Lymphoid Structure Scores in 7 Cohorts
eFigure 8. Prognostic Outcome of Tertiary Lymphoid Structure Score Across 3 Cancer Types in 4 Cohorts
eFigure 9. Kaplan-Meier Curves of Progression-Free Survival for Patients With High vs Low Overall Tertiary Lymphoid Structure (TLS) Scores vs No TLSs
eFigure 10. Kaplan-Meier Survival Analysis of Overall Survival by Individual Tertiary Lymphoid Structure 1-3 Score
eFigure 11. Concordance Index of Overall Survival Predictions Using Overall Tertiary Lymphoid Structure (TLS) Score and Individual TLS1-3 Scores
eFigure 12. Concordance Index of Overall Survival Predictions Using Tertiary Lymphoid Structure Score and Density
eFigure 13. Comparison of C Index for Predicting Overall Survival Using Tertiary Lymphoid Structure Score, Tumor Stage, and Combined Model
eFigure 14. Forest Plot of Tertiary Lymphoid Structure Score for The Cancer Genome Atlas Esophageal Carcinoma
eFigure 15. Forest Plot of Tertiary Lymphoid Structure Score for The Cancer Genome Atlas Stomach Adenocarcinoma
eFigure 16. Forest Plot of Tertiary Lymphoid Structure Score for Southern Medical University Stomach Adenocarcinoma
eFigure 17. Forest Plot of Tertiary Lymphoid Structure Score for The Cancer Genome Atlas Colon Adenocarcinoma
eFigure 18. Forest Plot of Tertiary Lymphoid Structure Score for The Cancer Genome Atlas Rectum Adenocarcinoma
eFigure 19. Forest Plot of Tertiary Lymphoid Structure Score for The Cancer Genome Atlas Liver Hepatocellular Carcinoma
eFigure 20. Forest Plot of Tertiary Lymphoid Structure Score for The Cancer Genome Atlas Pancreatic Adenocarcinoma
eFigure 21. Kaplan-Meier Survival Analysis by Tertiary Lymphoid Structure Score for Patients With Same Tumor Stage
eFigure 22. Comparison of Tertiary Lymphoid Structure Scores Calculated Using Weights Trained on Different Cohorts
eFigure 23. Correlation Between Tertiary Lymphoid Structure Scores and 10 Tumor

© 2023 Li Z et al. *JAMA Network Open*.

Microenvironmental Cell Types Estimated From Gene Expression Data in The Cancer Genome Atlas Stomach Adenocarcinoma Cohort
eFigure 24. Gene Expression Profile of 23 Cytokines Correlated With Tertiary Lymphoid Structure Score in The Cancer Genome Atlas Stomach Adenocarcinoma Cohort
eFigure 25. Molecular Signature of Tertiary Lymphoid Structure Score and Prognostic Value
eFigure 26. Multivariate Survival Analysis of Tertiary Lymphoid Structure Molecular Signature in Combined Gastric Cancer Gene Expression Data Sets
eFigure 27. Multivariate Survival Analysis of Tertiary Lymphoid Structure Molecular Signature in Combined Colorectal Cancer Gene Expression Data Sets
eTable 1. Association Between Tertiary Lymphoid Structure Score and Tumor Stage or Grade in 7 Cohorts
eTable 2. Interslide Variability of Tertiary Lymphoid Structure Scores
eTable 3. Univariate and Multivariate Survival Analysis of Individual Tertiary Lymphoid Structure Scores in 7 Cohorts and Combined Data Set
eTable 4. Univariate and Multivariate Survival Analysis of Overall Tertiary Lymphoid Structure Scores in The Cancer Genome Atlas Esophageal Carcinoma
eTable 5. Univariate and Multivariate Survival Analysis of Overall Tertiary Lymphoid Structure Scores in The Cancer Genome Atlas Stomach Adenocarcinoma
eTable 6. Univariate and Multivariate Survival Analysis of Overall Tertiary Lymphoid Structure Scores in Southern Medical University Stomach Adenocarcinoma
eTable 7. Univariate and Multivariate Survival Analysis of Overall Tertiary Lymphoid Structure Scores in The Cancer Genome Atlas Colon Adenocarcinoma
eTable 8. Univariate and Multivariate Survival Analysis of Overall Tertiary Lymphoid Structure Scores in The Cancer Genome Atlas Rectum Adenocarcinoma
eTable 9. Univariate and Multivariate Survival Analysis of Overall Tertiary Lymphoid Structure Scores in The Cancer Genome Atlas Liver Hepatocellular Carcinoma
eTable 10. Univariate and Multivariate Survival Analysis of Overall Tertiary Lymphoid Structure Scores in The Cancer Genome Atlas Pancreatic Adenocarcinoma
eTable 11. The 11 Cytokine Genes and Corresponding Weights in Tertiary Lymphoid Structure Molecular Signature

This supplemental material has been provided by the authors to give readers additional information about their work.

© 2023 Li Z et al. *JAMA Network Open*.

eMethods

Image preprocessing

All H&E images were reviewed to ensure sufficient image quality. Whenever available, images at the 40× magnification were processed and analyzed; 65 slides were scanned at 20× and the corresponding images were used. To minimize the influence of image artifacts, we used the Openslide software to down-sample the whole-slide images by a factor of 32, and then removed those regions with pen marks, folding and blurring artifacts by using appropriate color filters (https://github.com/histolab/histolab).

Tumor detection

Since only TLS within or around the tumor area are relevant, we first trained a deep learning model for automated tumor detection in histopathology images. For this purpose, we used publicly available and previously annotated H&E-stained whole-slide images of colorectal cancer and stomach cancer as the training dataset[1] (https://doi.org/10.5281/zenodo.2530789). A total of 94 whole-slide images from 81 patients were used to create 11977 image tiles of 512×512 at 0.5 μm/pixel. Each image tile was manually annotated as tumor and non-tumor (including adipose tissue, mucus, stroma or muscle). We used the ResNet18 deep learning model and adopted the same experimental setting described previously[2]. During the training process, we used horizontal/vertical flipping and translation to augment the training dataset. We employed a cross-entropy loss function and used the Adam optimizer with a learning rate of 5×10^{-6} for training, and counteracted overfitting by an L2-regularization of 1×10^{-4}. The batch size was 64 and training was run for 25 epochs. Finally, tumor segmentation was expanded via image dilation by 0.5 mm to include the invasive margin.

Nuclei segmentation and lymphocyte classification

For single-cell analysis, we trained a Mask R-CNN deep learning model to segment and classify individual nuclei into tumor cells, lymphocytes, and other nonmalignant cells. For this purpose, we used our previously curated public dataset set, which contains a total of manually annotated 17,582 tumor cells, 22,550 lymphocytes, and 10,675 other non-malignant cells in 1358 image patches from 66 patients in the TCGA-LIHC dataset (https://github.com/zilanjiuwan/Single-Cell-Imaging-Analysis-of-HCC-Data). We adopted the same experimental setting described in our previous study[3]. Specifically, we used horizontal/vertical flipping, scaling, rotation, contrast normalization, affine transformation, and Gaussian blurring to augment

© 2023 Li Z et al. *JAMA Network Open*.

the training dataset. We employed a stochastic gradient decent with momentum of 0.9, a weight decay of 1×10^{-4}, and a batch size of 4. The network was trained for 20,000 iterations, starting from a learning rate of 0.001, and reducing to 0.0002 at 16,000 and 0.0001 at 18,000 iterations. The Mask R-CNN model was trained in TensorFlow and Keras platform and trained using the NVIDIA Tesla V100 (32GB).

In addition to TLS scoring, we also computed the density of tumor-infiltrating lymphocytes (excluding TLS) per tumor area based on the results of nuclei segmentation and classification.

Molecular evaluation of the imaging-based TLS scores

We used the matched histopathology image and gene expression data in TCGA cohorts to explore the underlying molecular features associated with the TLS scores computed on images. Since TLS consists of multiple cell types including B and T lymphocytes, we assessed the correlation between TLS scores and the abundance of immune cell infiltrate estimated from gene expression data. In addition, because cytokines play a critical role in mediating the formation and maturation of TLS, we also assessed the relations between TLS scores and cytokine gene expression levels. We further developed a gene signature for the imaging-based TLS score using the TCGA-STAD cohort as the training set. Finally, we evaluated the prognostic effect of the TLS gene signature in gastric and colorectal cancers given their high incidence rates among GI cancers. These cohorts include TCGA-STAD, TCGA-COAD/READ, and 6 additional largest datasets for which gene expression and survival data are publicly available (gastric cancer: GSE62254, GSE84437 and GSE15459; colorectal cancer: GSE39582, GSE14333 and GSE37892).

Genes associated with the TLS scores

We used the gene expression data and TLS scores available for 335 patients in the TCGA-STAD cohort for this analysis. The absolute abundance of 10 different cell types in the TME were estimated from bulk gene expression data using the MCPcounter algorithm. The cytokine genes in the Cytokine-cytokine receptor interaction pathway of the Kyoto Encyclopedia of Genes and Genomes (KEGG) database were included in the following analysis. One-hundred and four cytokine genes were measured in all the patients. All the features were centered and scaled. Due to high percentage of patients with no detectable TLS, we applied a univariate tobit model (R package VGAM), which was specifically designed to model zero-inflated data to assess the correlations between cytokines or immune cell abundance with the imaging-based TLS score.

© 2023 Li Z et al. *JAMA Network Open*.

Development of a gene expression signature of TLS score

We used the TCGA-STAD cohort as the training set given that it had the highest levels of TLS. Considering that different types of TLS (1 vs. 2 vs. 3) are biologically related and may share common molecular regulatory mechanisms, instead of modeling each TLS separately, we employed a multi-task learning scheme to conduct feature selection and model construction to predict the 3 TLS scores simultaneously. First, we used the univariate tobit model to assess the correlations between cytokines with each of the 3 TLS scores. The resulting P values for each gene was adjusted using Benjamini & Hochberg method. We selected cytokines that were significantly associated with two or all the TLS scores (FDR < 0.1) as the candidate genes. Based on these features, we trained a multi-task linear regression model in the TCGA-STAD cohort (R package, RMTL), with 'L21' norm and penalty strength determined by 10-fold cross validation. In the final model, 11 cytokines were included with non-zero weights associated with each of the 3 TLS scores. The gene signature for the overall TLS score was calculated based on the weights determined previously.

Validation of the TLS gene signature in independent cohorts

The prognostic effect of gene signature of TLS score was assessed in 8 large independent cohorts of gastric or colorectal cancers. Overall survival information is available for all the gastric cohorts and was used to assess the prognostic effect of TLS gene signature. Recurrence free survival is available for all the colorectal cohorts and its relationship with gene signature was assessed. The combined prognostic effect in multiple cohorts was estimated using the generic inverse variance method (R package meta). Clinicopathologic variables such as age, gender, tumor stage, and the estimated abundance of CTLs were included in the multivariate Cox regression analysis.

© 2023 Li Z et al. *JAMA Network Open*.

eResults

Different weighting of individual TLS scores

We then investigated different weighting of individual TLS scores. Instead of using TCGA-STAD as the training set, we retrained a linear model using the SMU-STAD, TCGA-PAAD, or combined dataset. The overall TLS scores remained highly stable with Pearson correlation >0.93 and prognostic patterns were similar to the original results (**eFig**. 22).

Molecular correlates and gene signature of TLS score

We investigated the molecular features related to TLS and set out to develop a gene signature for TLS scores. Among the 10 immune and stromal cell types estimated from gene expression data, score for TLS 1 had the strongest correlations with T cell abundance including CD8 T cells, while score for TLS 2 was most correlated with B cell abundance. The score for TLS 3 had strong positive correlations with both T and B cells, consistent with the enrichment of both cell types in mature TLS (**eFig. 23**). All TLS scores had negative correlations with neutrophils and fibroblasts, which often play an immunosuppressive role in the tumor microenvironment. Among 104 cytokine genes, we identified 23 cytokines that were significantly correlated with the imaging-based TLS score (**eFig. 24**). Considering that the TCGA-STAD cohort had the highest level of TLS compared with other cancer types, we used this cohort as the training set to obtain a gene signature for TLS score (**eTable 11**). The final model consists of 11 cytokine genes: 5 genes including *CXCL13, CXCL11, CXCL10, CD40LG, LTA* had positive weights and were upregulated in tumors with a high TLS score; while 6 genes including *INHBB, INHBA, TGFB2, VEGFB, PDGFA, CLCF1* had negative weights and were downregulated in tumors with a high TLS score (**eFig. 25A**). There was significant association ($P < 0.0001$ and $P = 0.00138$) between the 11-gene signature and TLS score in TCGA-STAD and TCGA-COAD/READ cohorts.

We finally assessed the prognostic effect of the 11-gene signature of TLS in two most common GI cancers, i.e., gastric and colorectal cancers (e**Fig. 25B-C**). We observed a consistently favorable prognostic effect of the TLS gene signature in 4 independent cohorts of gastric cancer, with overall HR = 0.75 (95% CI: 0.7 to 0.81, $P < 0.001$). Similarly, the 11-gene signature also had a favorable prognostic effect in 4 independent cohorts of colorectal cancer with overall HR = 0.68 (95% CI: 0.61 to 0.75, $P < 0.001$). In multivariable analysis, the TLS gene signature remained an independent prognostic factor when adjusting for clinicopathologic factors and abundance of cytotoxic lymphocytes in both gastric cancer (HR = 0.74, 95% CI: 0.68−0.80, $P < 0.001$) and colorectal cancer (HR = 0.78, 95% CI: 0.70−0.87, $P < 0.001$), as shown in **eFig. 26-27**.

© 2023 Li Z et al. *JAMA Network Open*.

eReferences

1. Kather JN, Krisam J, Charoentong P, et al. Predicting survival from colorectal cancer histology slides using deep learning: A retrospective multicenter study. *Plos Medicine* 2019; **16**(1).
2. Kather JN, Pearson AT, Halama N, et al. Deep learning can predict microsatellite instability directly from histology in gastrointestinal cancer. *Nat Med* 2019; **25**(7): 1054-6.
3. Wang H, Jiang Y, Li B, Cui Y, Li D, Li R. Single-Cell Spatial Analysis of Tumor and Immune Microenvironment on Whole-Slide Image Reveals Hepatocellular Carcinoma Subtypes. *Cancers (Basel)* 2020; **12**(12).

© 2023 Li Z et al. *JAMA Network Open*.

eFigures. Supplementary Figures

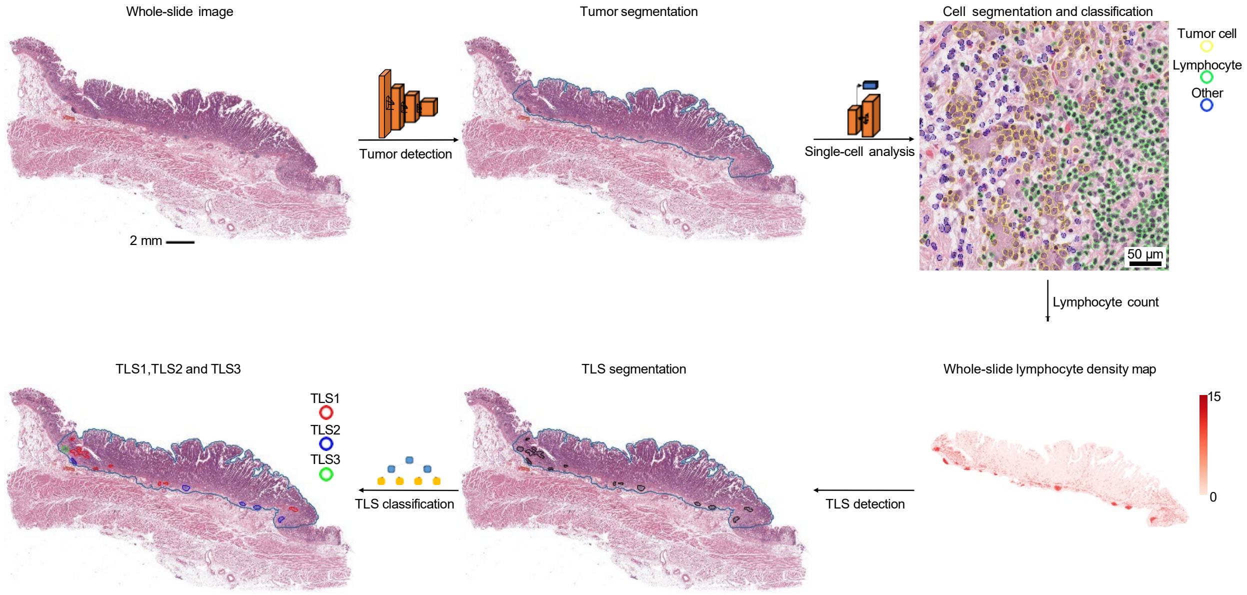

eFigure 1. Proposed Workflow for Automated Tertiary Lymphoid Structure Evaluation on Hematoxylin-Eosin–Stained Whole-Slide Images

Computational pipeline for artificial intelligence-based detection, classification, and quantitative evaluation of TLS. Our computational pipeline uses whole-slide images (WSIs) as input and consists of three modules: (1) a ResNet18 model for segmenting tumor areas in WSIs, (2) a Mask RCNN model for segmenting lymphocytes in tumor areas, and (3) the classification and regression trees (CART) algorithm for classifying individual TLSs.

© 2023 Li Z et al. *JAMA Network Open*.

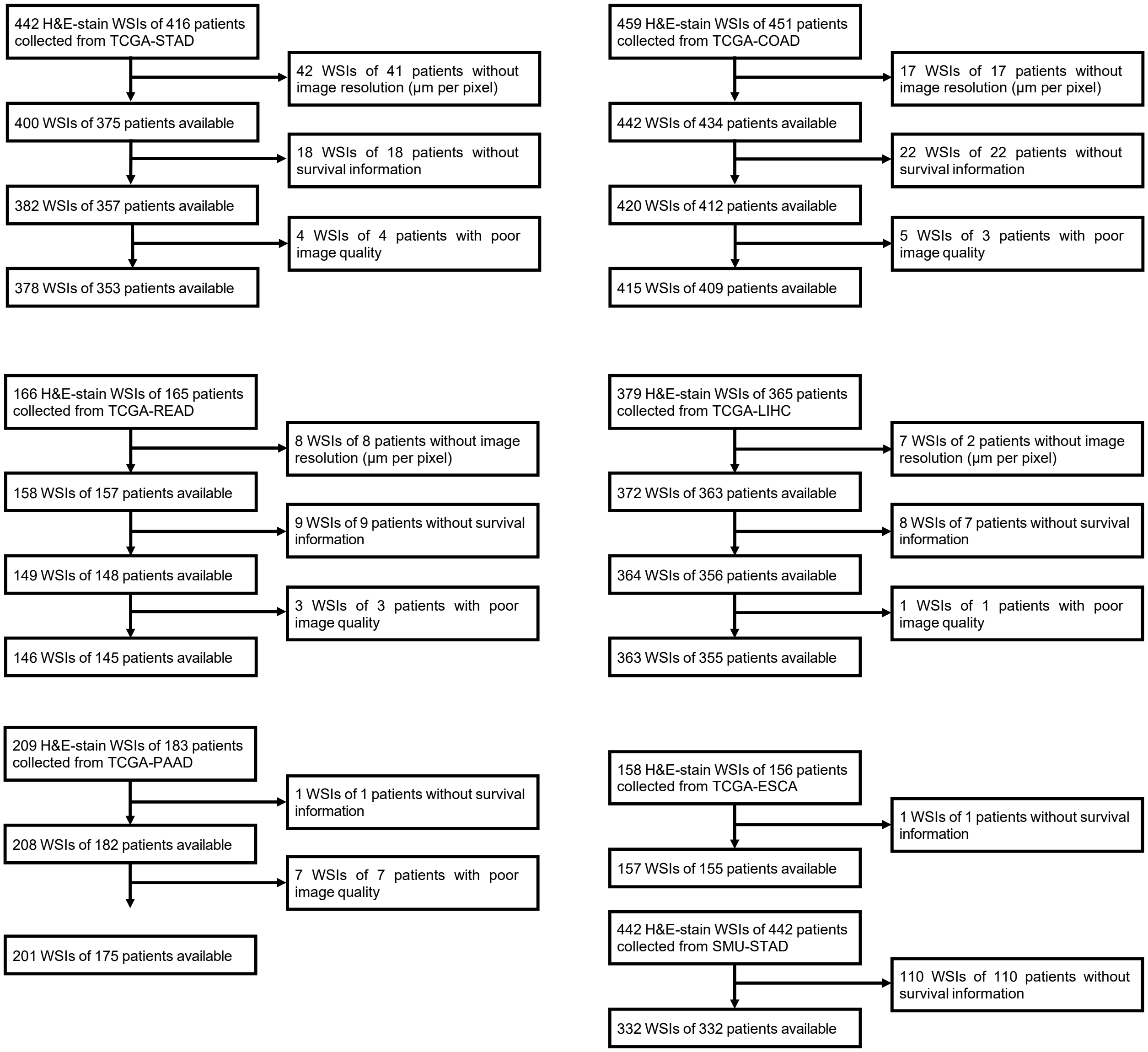

eFigure 2. Flow Chart of Patient Inclusion and Exclusion

© 2023 Li Z et al. *JAMA Network Open*.

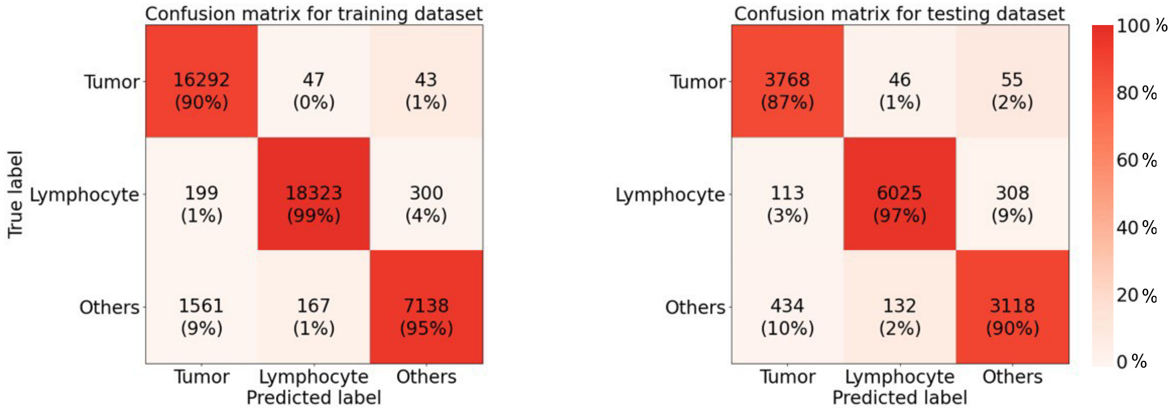

eFigure 3. Confusion Matrices for Nuclei Classification on Training and Testing Data Set

Values are the percentage and number of nuclei correctly and incorrectly classified by the Mask R-CNN model

© 2023 Li Z et al. *JAMA Network Open*.

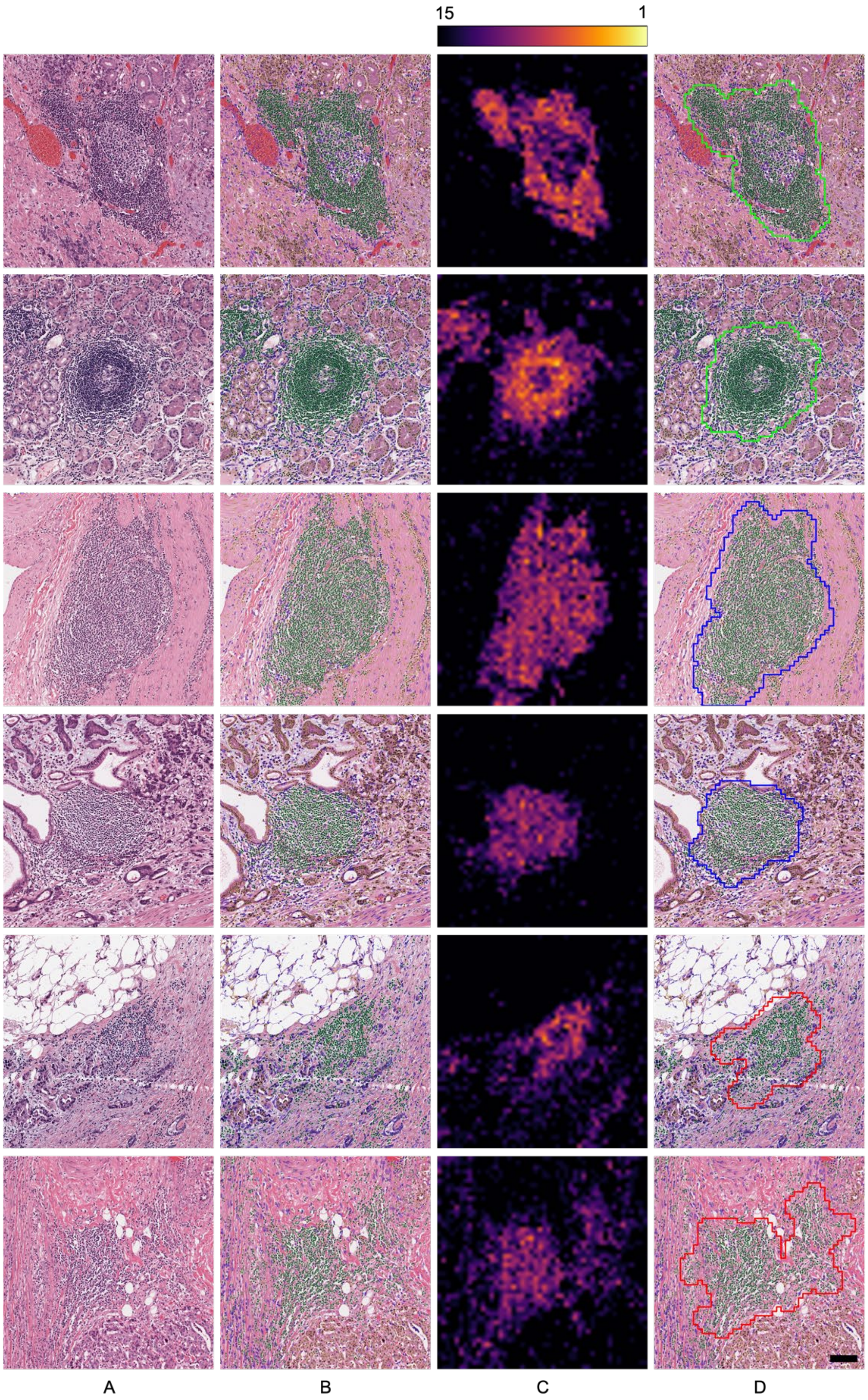

eFigure 4. Example Images for Tertiary Lymphoid Structure Segmentation and Classification

Example image patches (A) with cell segmentation and classification (B), lymphocyte density maps (C) and TLS segmentation and classification (D). Red, blue, and green outlines correspond to TLS1, TLS2 and TLS3, respectively. Scale bar: 100 μm.

© 2023 Li Z et al. *JAMA Network Open*.

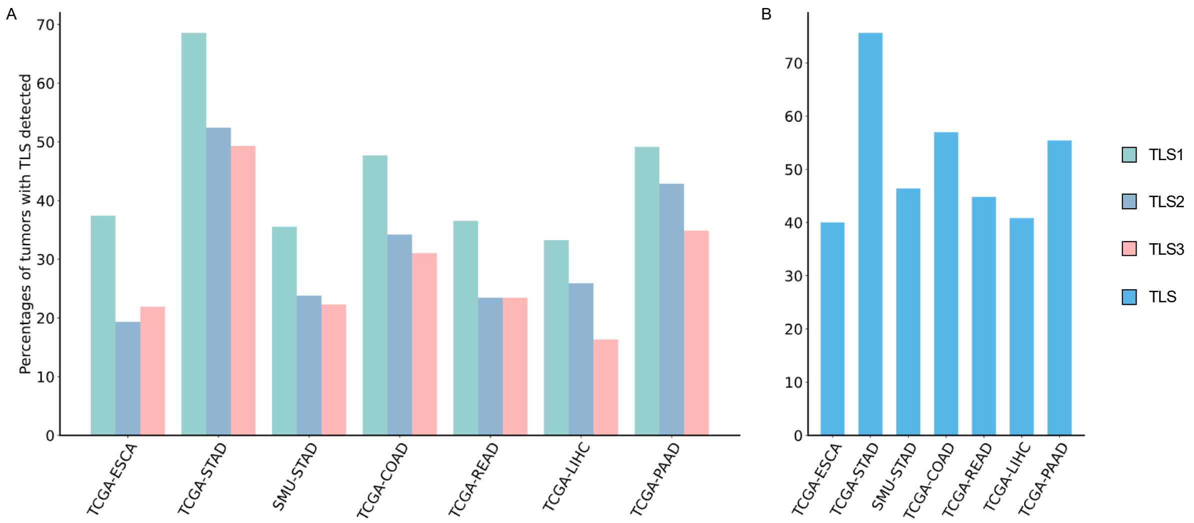

eFigure 5. Distributions of Tertiary Lymphoid Structure Across 6 Cancer Types in 7 Cohorts

The percentage of tumors in which TLS1-3 were detected (A) and any TLS were detected (B).

© 2023 Li Z et al. *JAMA Network Open*.

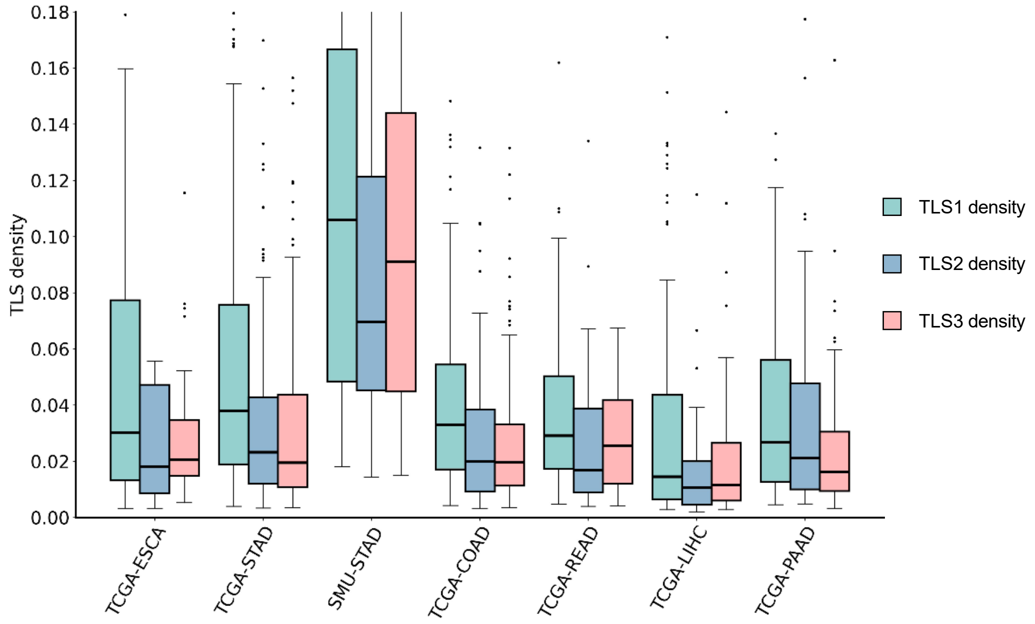

eFigure 6. Distributions of Tertiary Lymphoid Structure Density in 7 Cohorts

TLS density is defined as the number of TLS per unit tumor area. Patients with no TLS were excluded. All boxplots show median values, first and third quartiles; while whiskers extend to 1.5× the interquartile range.

© 2023 Li Z et al. *JAMA Network Open*.

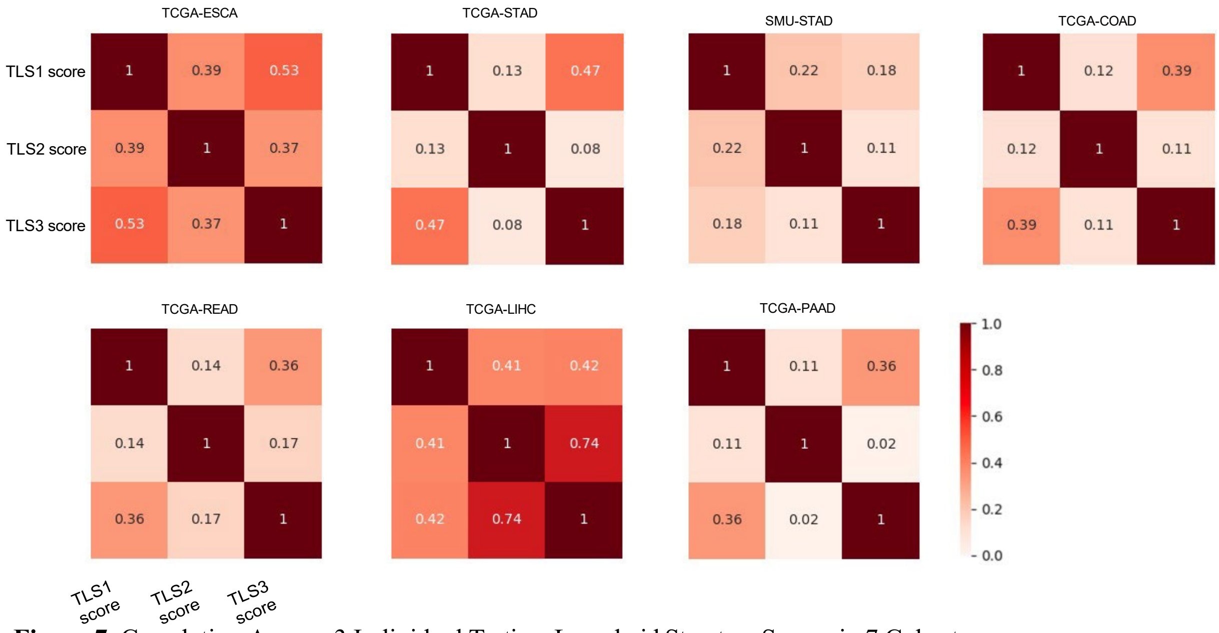

eFigure 7. Correlation Among 3 Individual Tertiary Lymphoid Structure Scores in 7 Cohorts
Values are Pearson correlation.

© 2023 Li Z et al. *JAMA Network Open*.

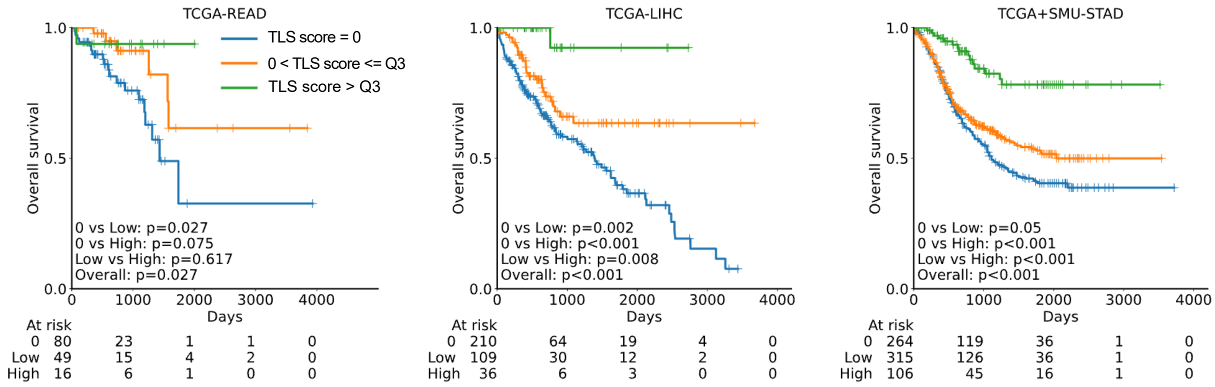

eFigure 8. Prognostic Outcome of Tertiary Lymphoid Structure Score Across 3 Cancer Types in 4 Cohorts

Kaplan-Meier curves of overall survival for patients with high vs low overall TLS scores vs no TLS. P values were determined by two-side log-rank test. Q3, upper quartile.

© 2023 Li Z et al. *JAMA Network Open*.

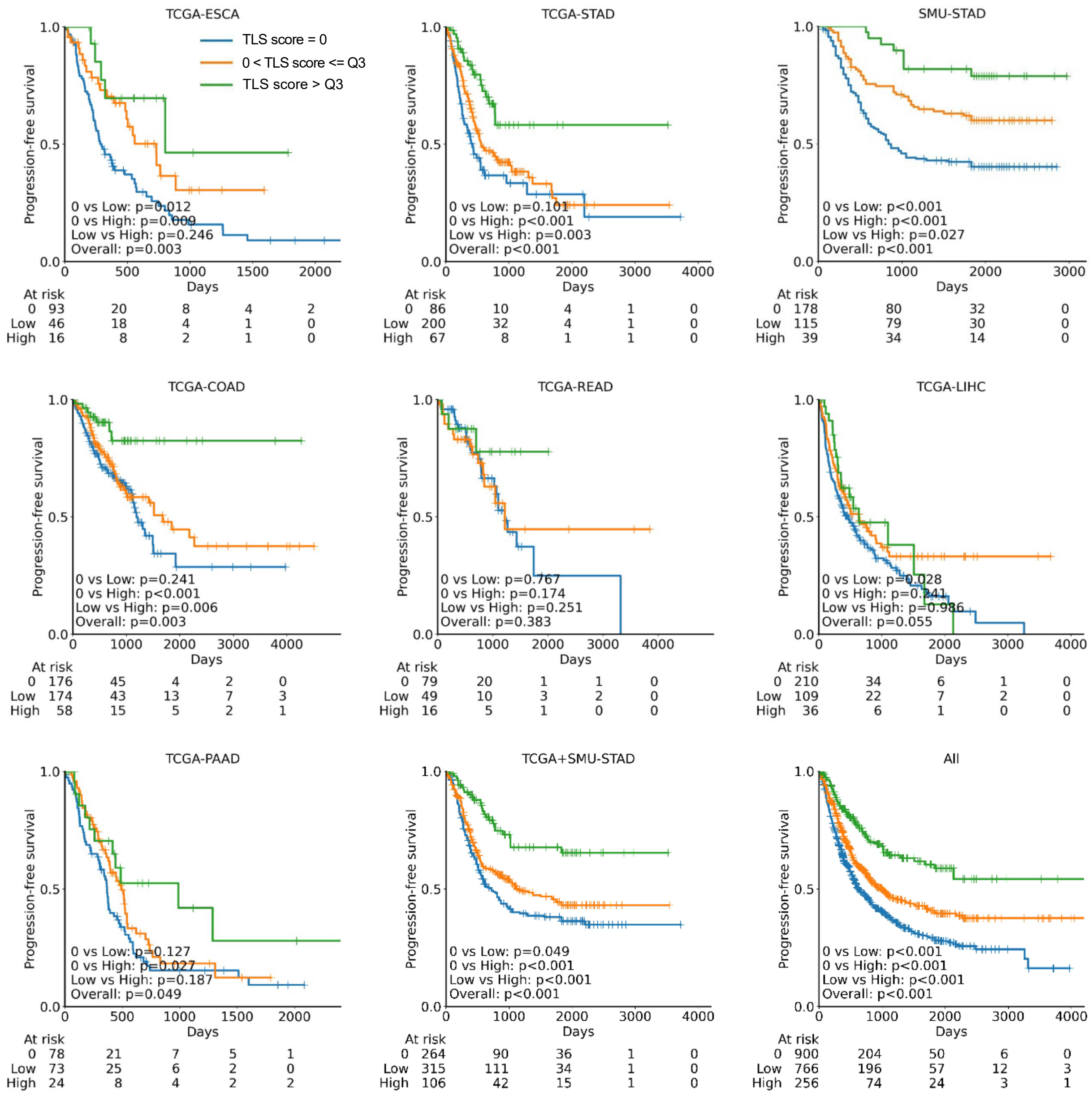

eFigure 9. Kaplan-Meier Curves of Progression-Free Survival for Patients With High vs Low Overall Tertiary Lymphoid Structure (TLS) Scores vs No TLSs

P values were determined by two-side log-rank test. Q3, upper quartile.

© 2023 Li Z et al. *JAMA Network Open*.

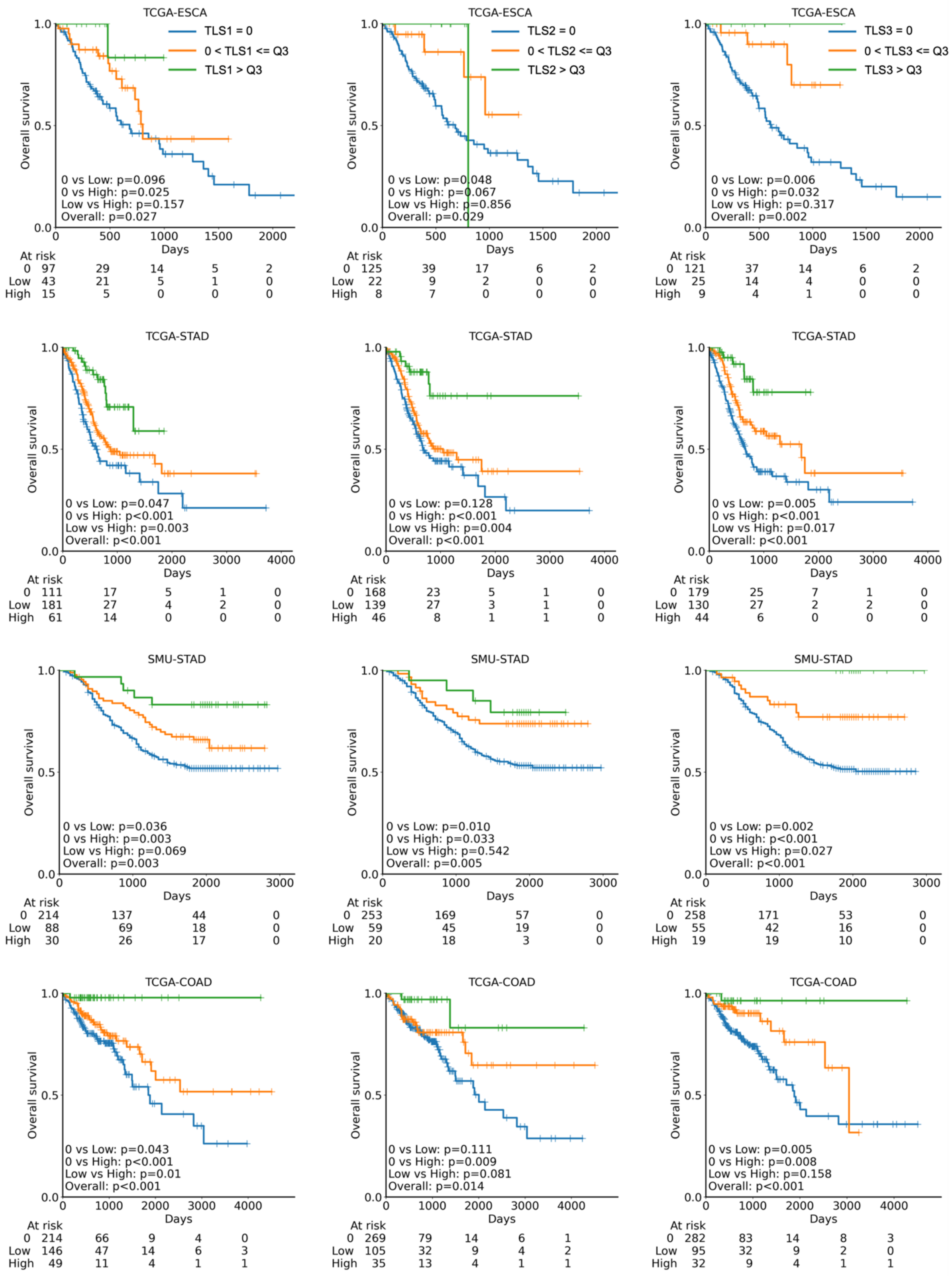

© 2023 Li Z et al. *JAMA Network Open*.

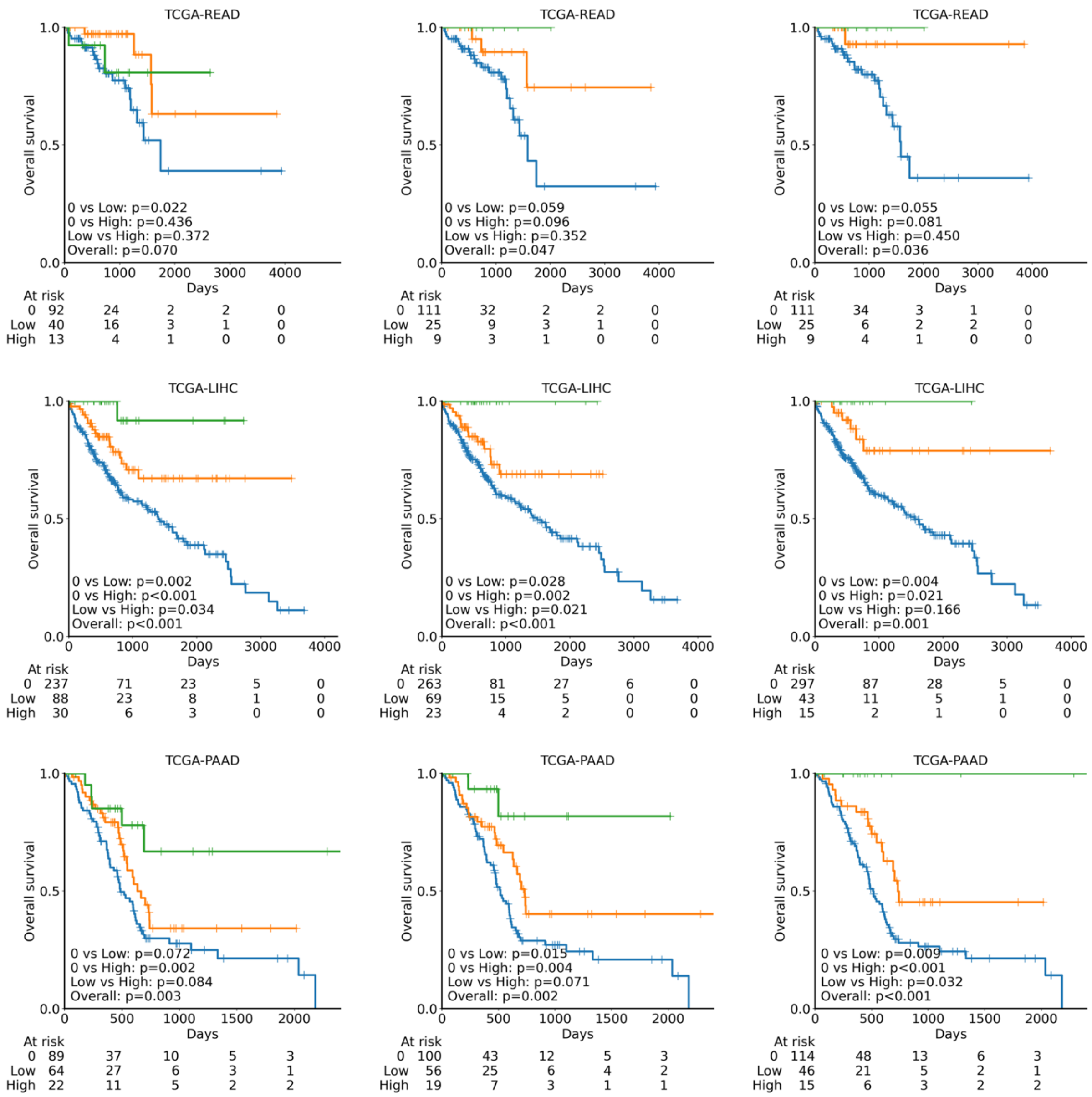

eFigure 10. Kaplan-Meier Survival Analysis of Overall Survival by Individual Tertiary Lymphoid Structure 1-3 Score

P values were determined by two-side log-rank test. Q3, upper quartile.

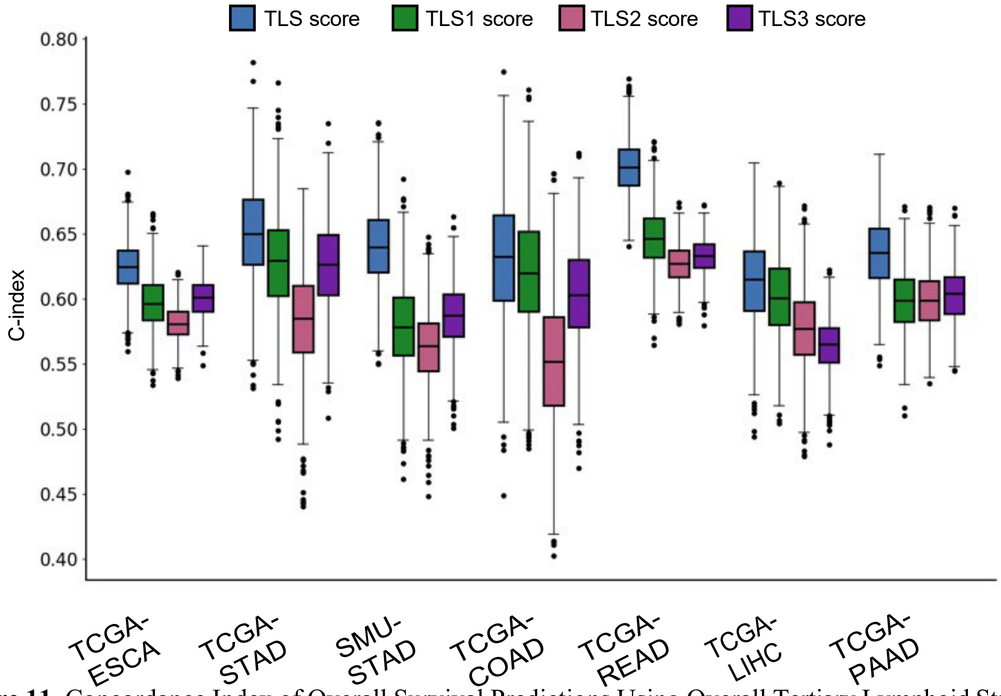

eFigure 11. Concordance Index of Overall Survival Predictions Using Overall Tertiary Lymphoid Structure (TLS) Score and Individual TLS1-3 Scores

Each boxplot (n=100) was calculated by bootstrap with 1000 repetitions. All boxplots contain quartiles and median values. whiskers extend to 1.5× the interquartile range.

© 2023 Li Z et al. *JAMA Network Open*.

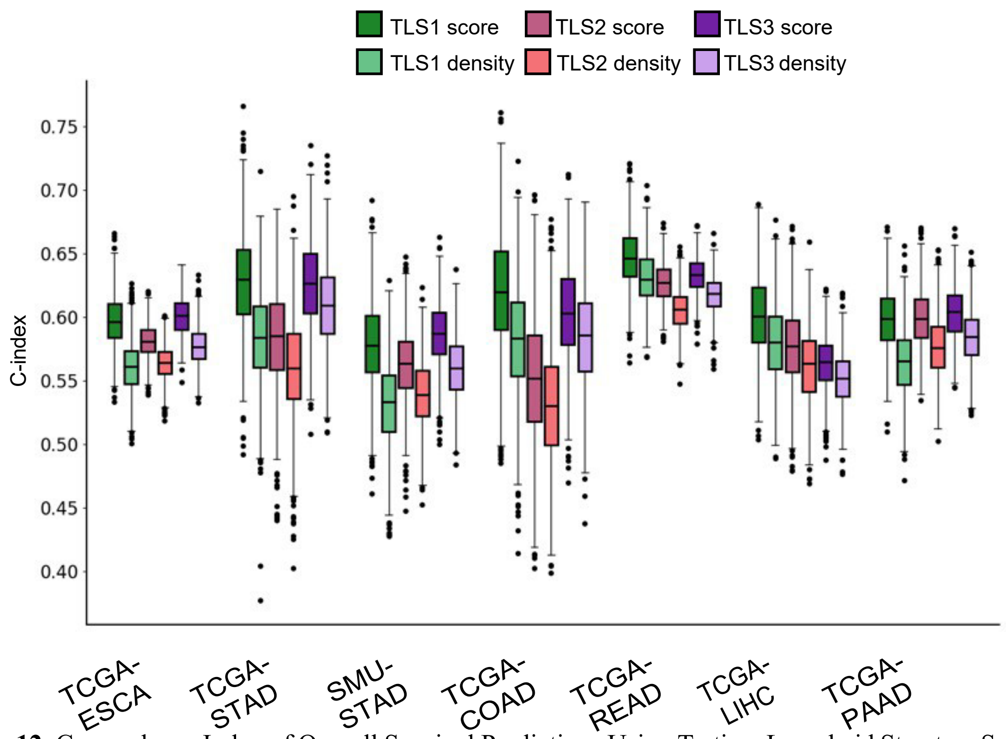

eFigure 12. Concordance Index of Overall Survival Predictions Using Tertiary Lymphoid Structure Score and Density

Each boxplot (n=100) was calculated by bootstrap with 1000 repetitions. All boxplots contain quartiles and median values. whiskers extend to 1.5× the interquartile range.

© 2023 Li Z et al. *JAMA Network Open*.

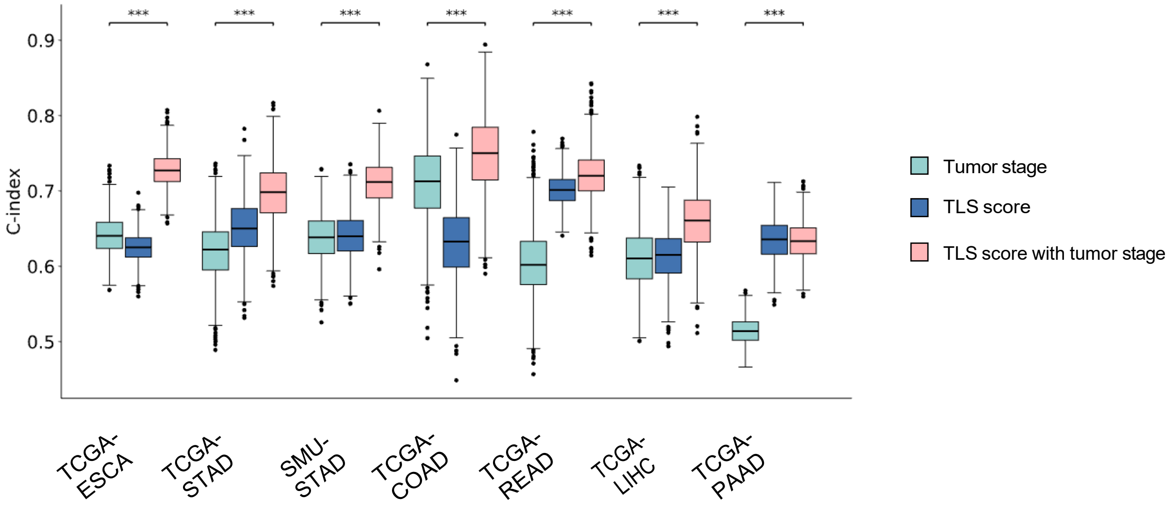

eFigure 13. Comparison of *C* Index for Predicting Overall Survival Using Tertiary Lymphoid Structure Score, Tumor Stage, and Combined Model

Each boxplot (n=100) was calculated by bootstrap with 1000 repetitions. All boxplots contain quartiles and median values. whiskers extend to 1.5× the interquartile range. P values were determined by two-side Mann-Whitney test. ***, $p < 0.001$.

© 2023 Li Z et al. *JAMA Network Open*.

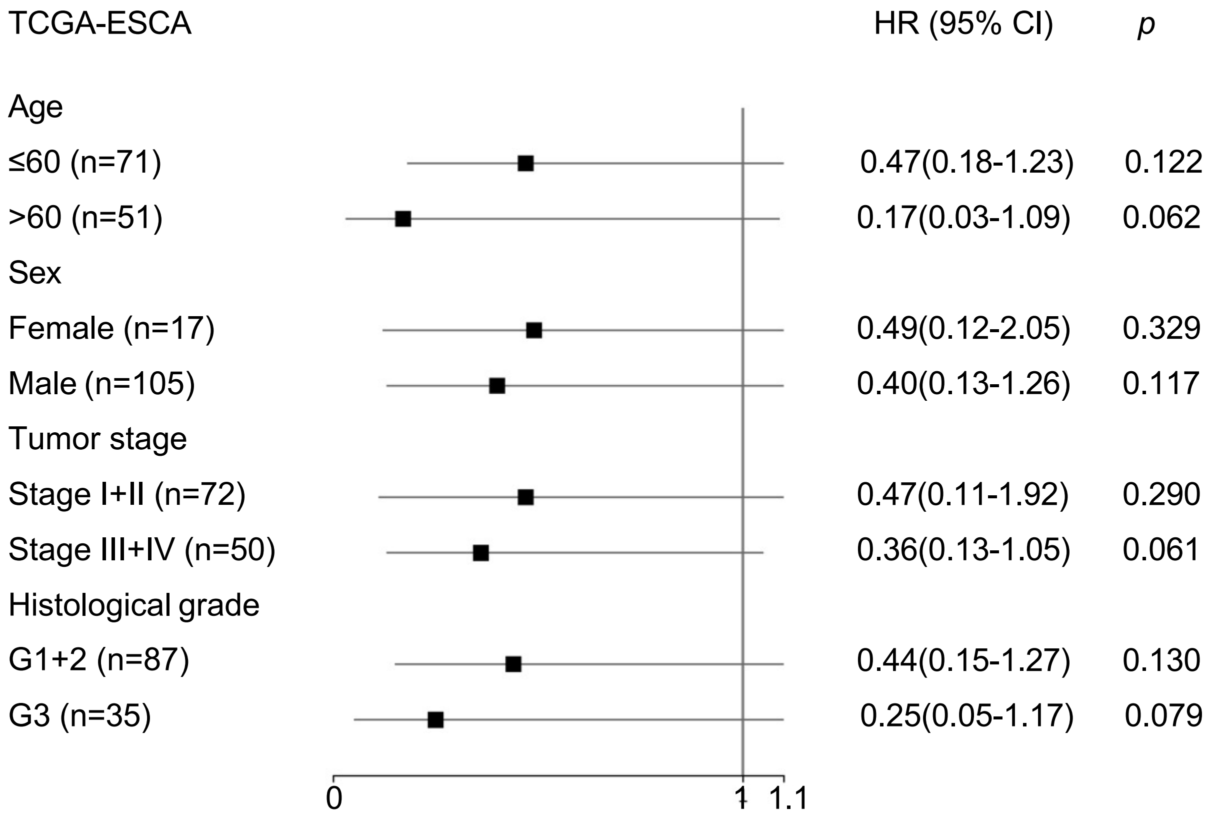

eFigure 14. Forest Plot of Tertiary Lymphoid Structure Score for The Cancer Genome Atlas Esophageal Carcinoma

Cox regression analysis was performed using the TLS score within each subgroup of patients.

© 2023 Li Z et al. *JAMA Network Open*.

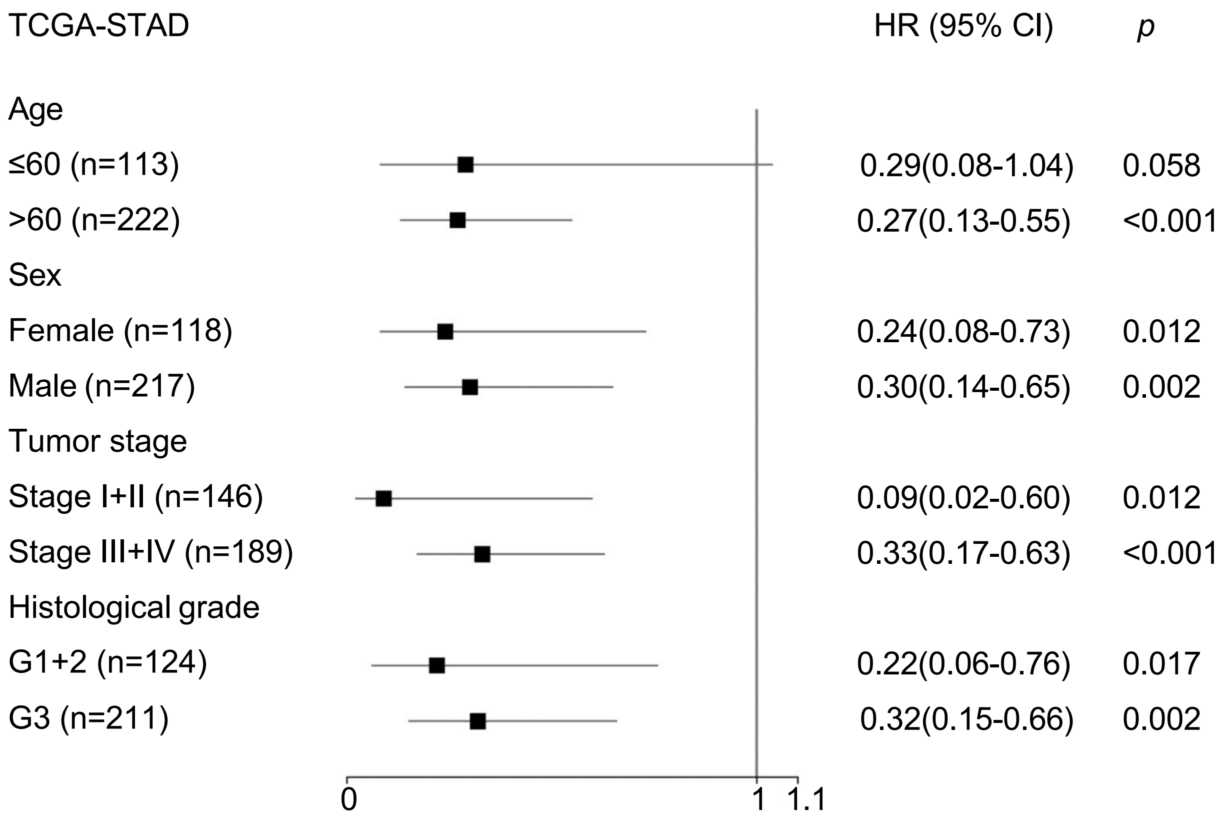

eFigure 15. Forest Plot of Tertiary Lymphoid Structure Score for The Cancer Genome Atlas Stomach Adenocarcinoma

Cox regression analysis was performed using the TLS score within each subgroup of patients.

© 2023 Li Z et al. *JAMA Network Open*.

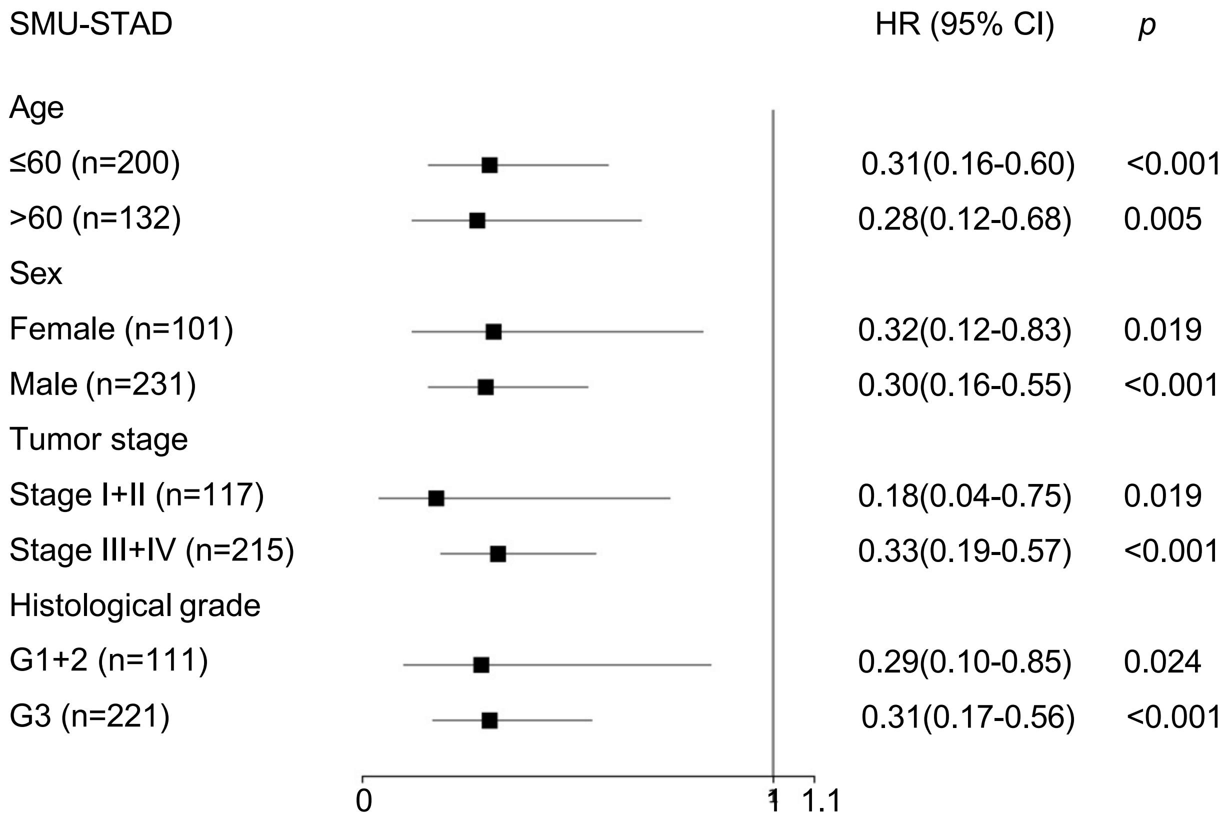

eFigure 16. Forest Plot of Tertiary Lymphoid Structure Score for Southern Medical University Stomach Adenocarcinoma

Cox regression analysis was performed using the TLS score within each subgroup of patients.

© 2023 Li Z et al. *JAMA Network Open*.

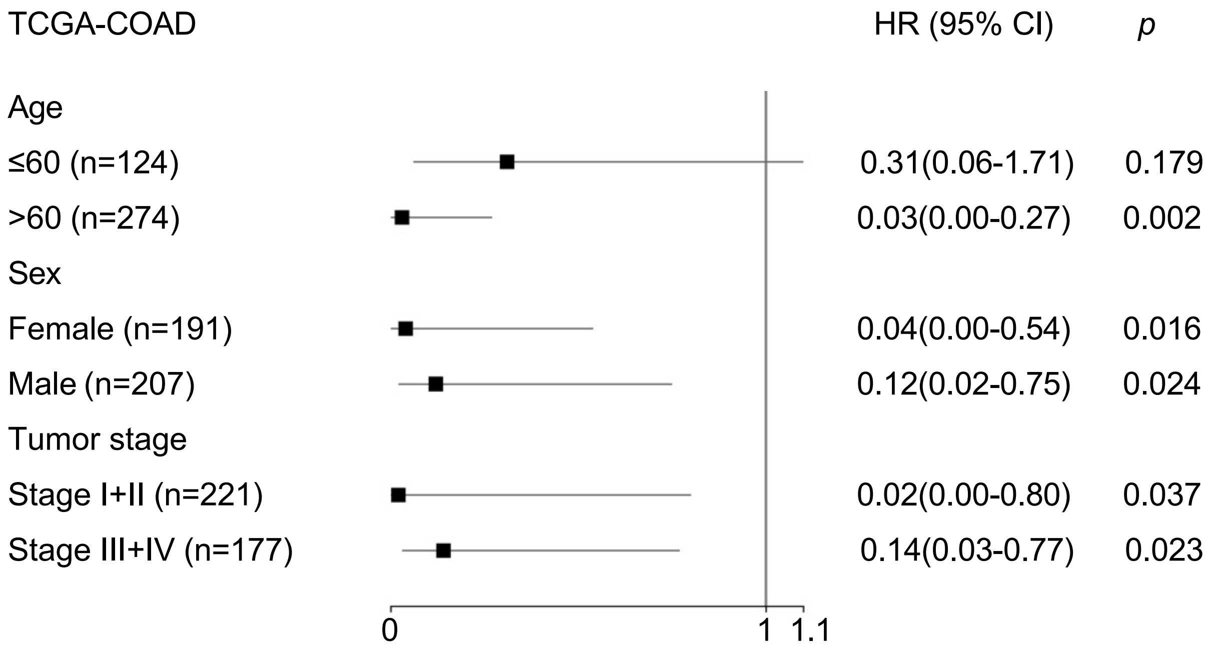

eFigure 17. Forest Plot of Tertiary Lymphoid Structure Score for The Cancer Genome Atlas Colon Adenocarcinoma

Cox regression analysis was performed using the TLS score within each subgroup of patients.

© 2023 Li Z et al. *JAMA Network Open*.

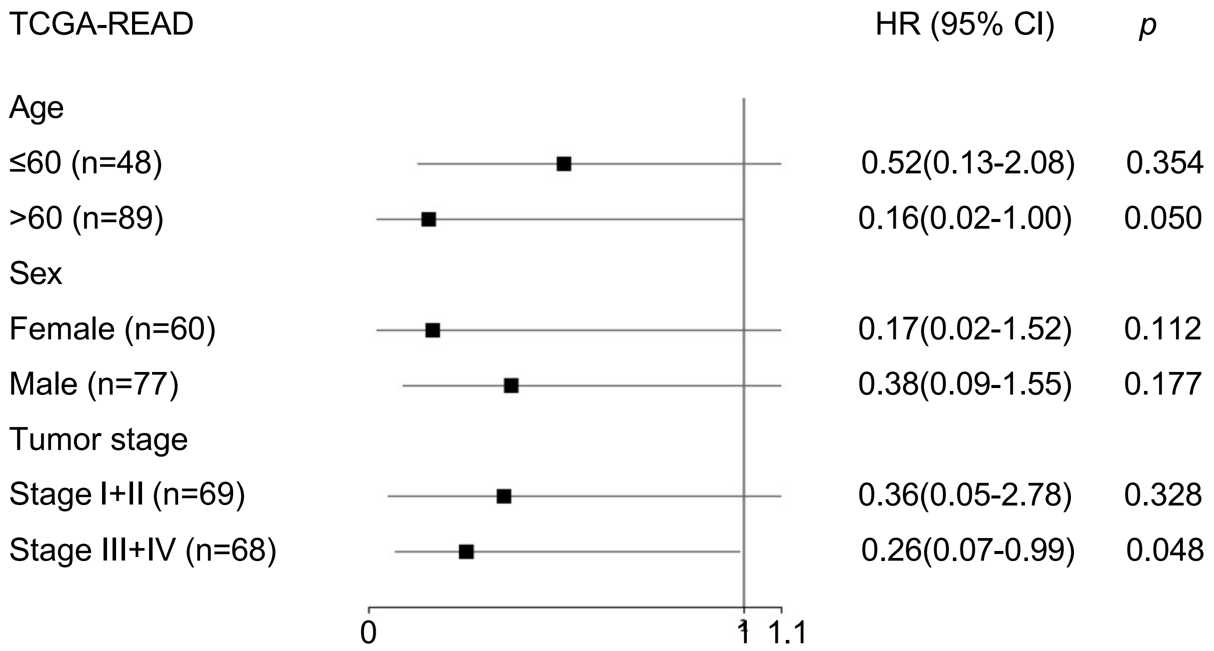

eFigure 18. Forest Plot of Tertiary Lymphoid Structure Score for The Cancer Genome Atlas Rectum Adenocarcinoma

Cox regression analysis was performed using the TLS score within each subgroup of patients.

© 2023 Li Z et al. *JAMA Network Open*.

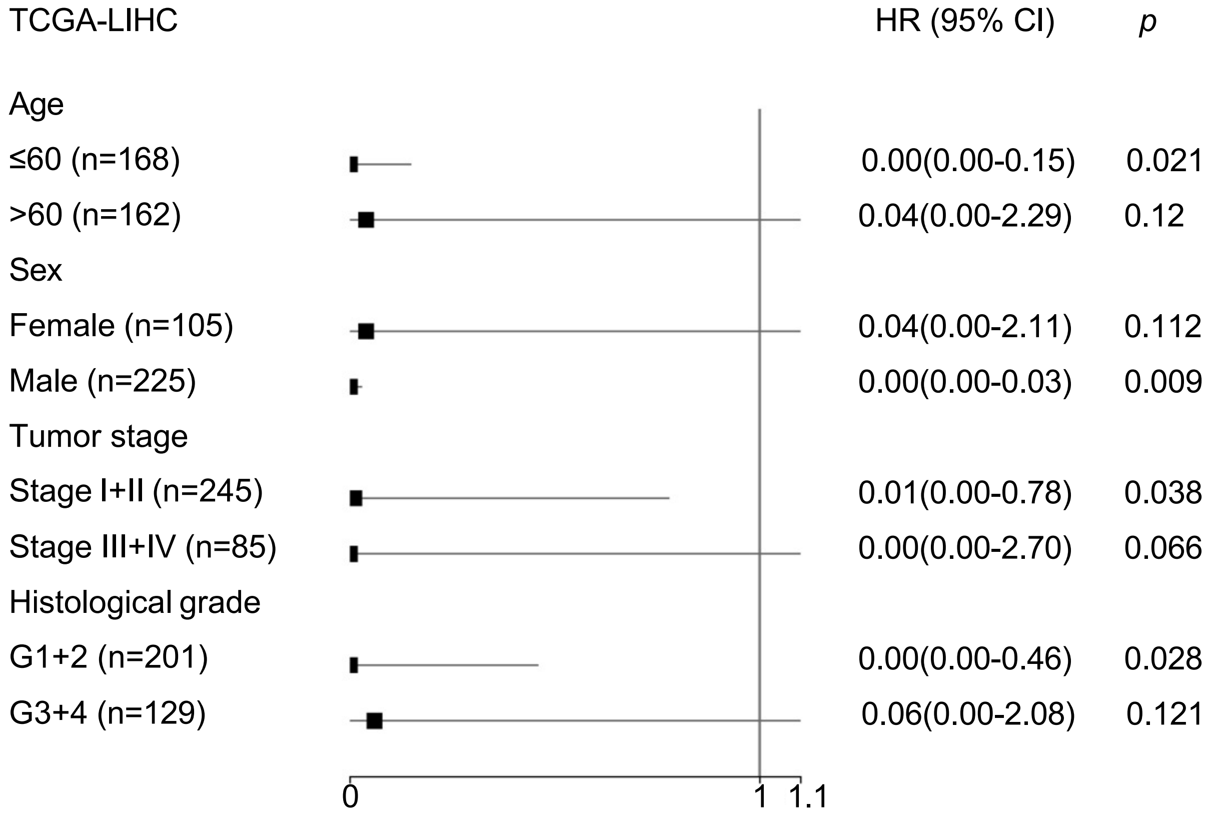

eFigure 19. Forest Plot of Tertiary Lymphoid Structure Score for The Cancer Genome Atlas Liver Hepatocellular Carcinoma

Cox regression analysis was performed using the TLS score within each subgroup of patients.

© 2023 Li Z et al. *JAMA Network Open*.

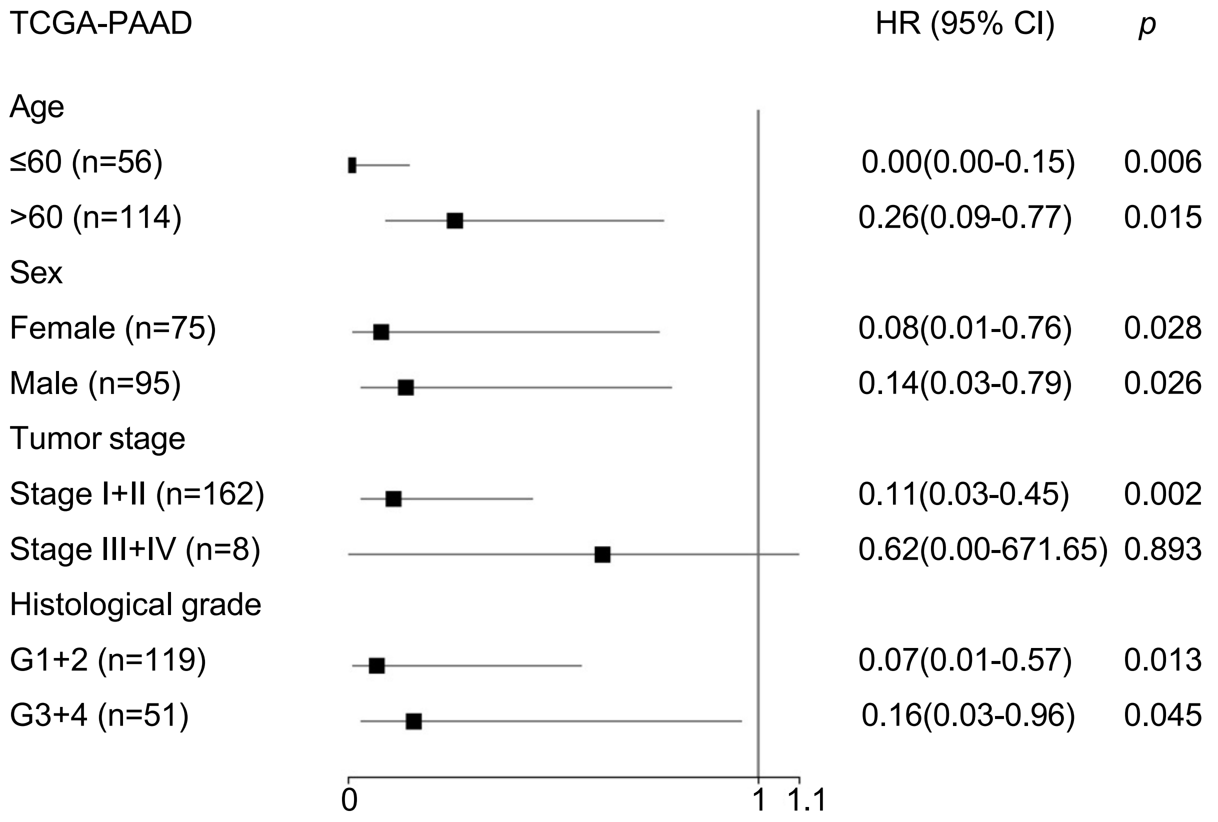

eFigure 20. Forest Plot of Tertiary Lymphoid Structure Score for The Cancer Genome Atlas Pancreatic Adenocarcinoma

Cox regression analysis was performed using the TLS score within each subgroup of patients.

© 2023 Li Z et al. *JAMA Network Open*.

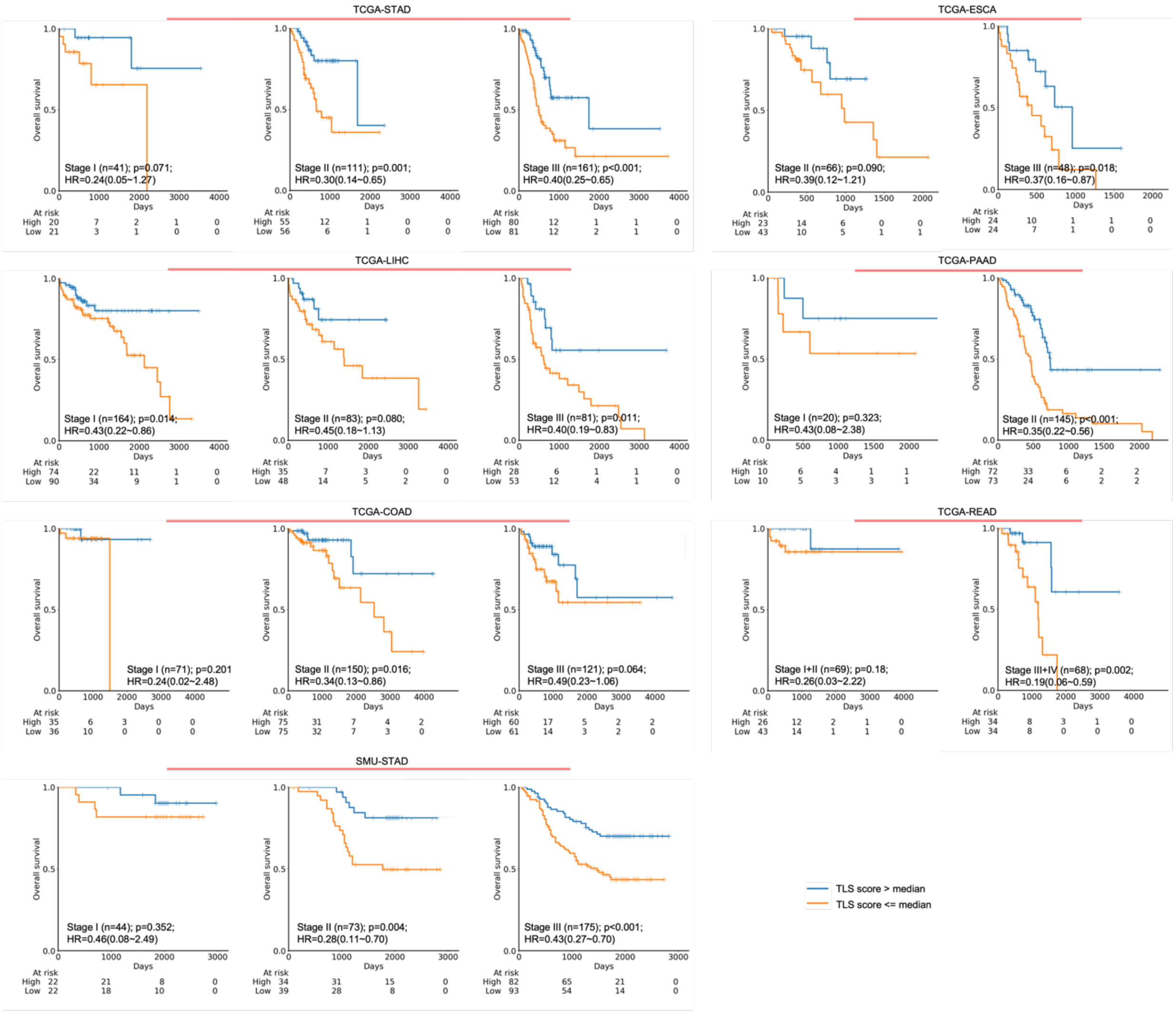

eFigure 21. Kaplan-Meier Survival Analysis by Tertiary Lymphoid Structure Score for Patients With Same Tumor Stage

P values were determined by two-sided log-rank test. Hazard ratios (HR) with 95% confidence interval were computed using the Cox proportional hazards model.

© 2023 Li Z et al. *JAMA Network Open*.

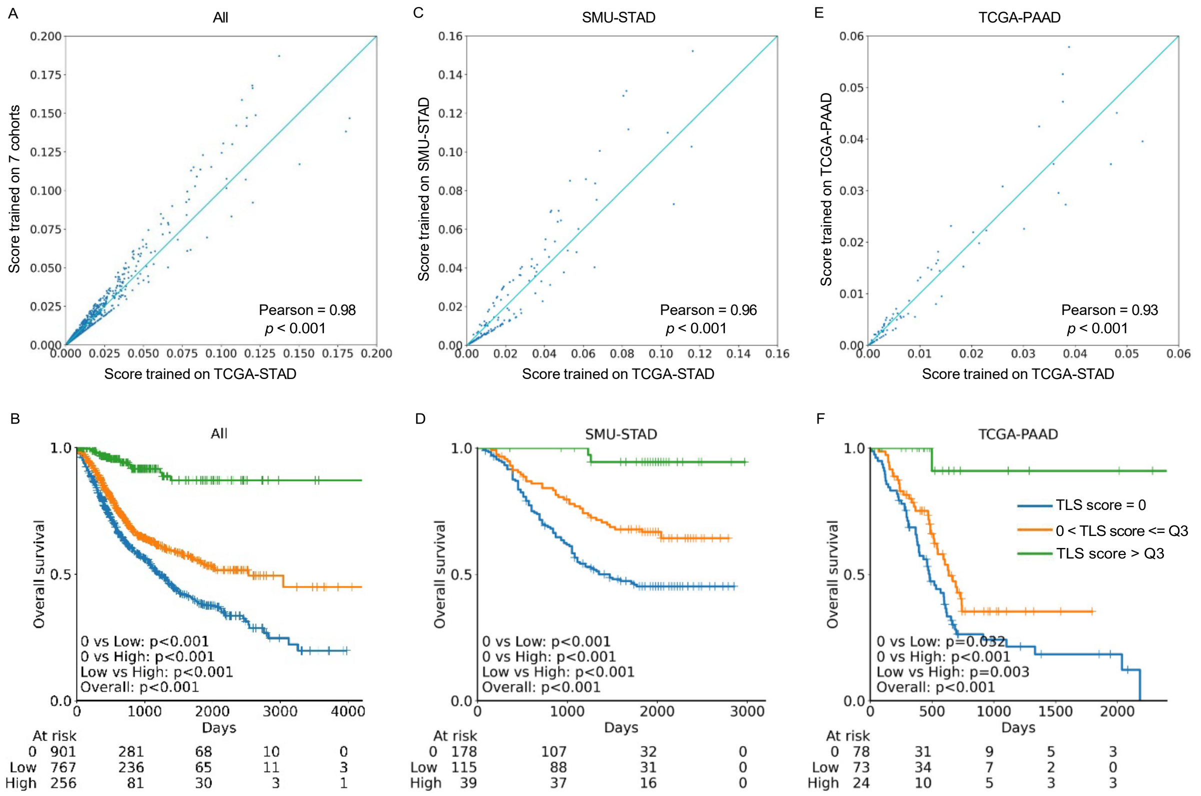

eFigure 22. Comparison of Tertiary Lymphoid Structure Scores Calculated Using Weights Trained on Different Cohorts

The x axis of each point (patient) in the 7 cohorts (A), SMU-STAD (C), and TCGA-PAAD (E) is the score calculated from the weights trained on TCGA-STAD. The y axis of each point (patient) is the score calculated from the weights trained on themselves. Values are the pair-wise Pearson correlation. Kaplan-Meier survival analysis of overall survival for patients with high vs low overall TLS scores vs no TLS in the 7 cohorts (B), SMU-STAD (D), and TCGA-PAAD

(F) using the weights trained on themselves, respectively. Q3, upper quartile.

© 2023 Li Z et al. *JAMA Network Open*.

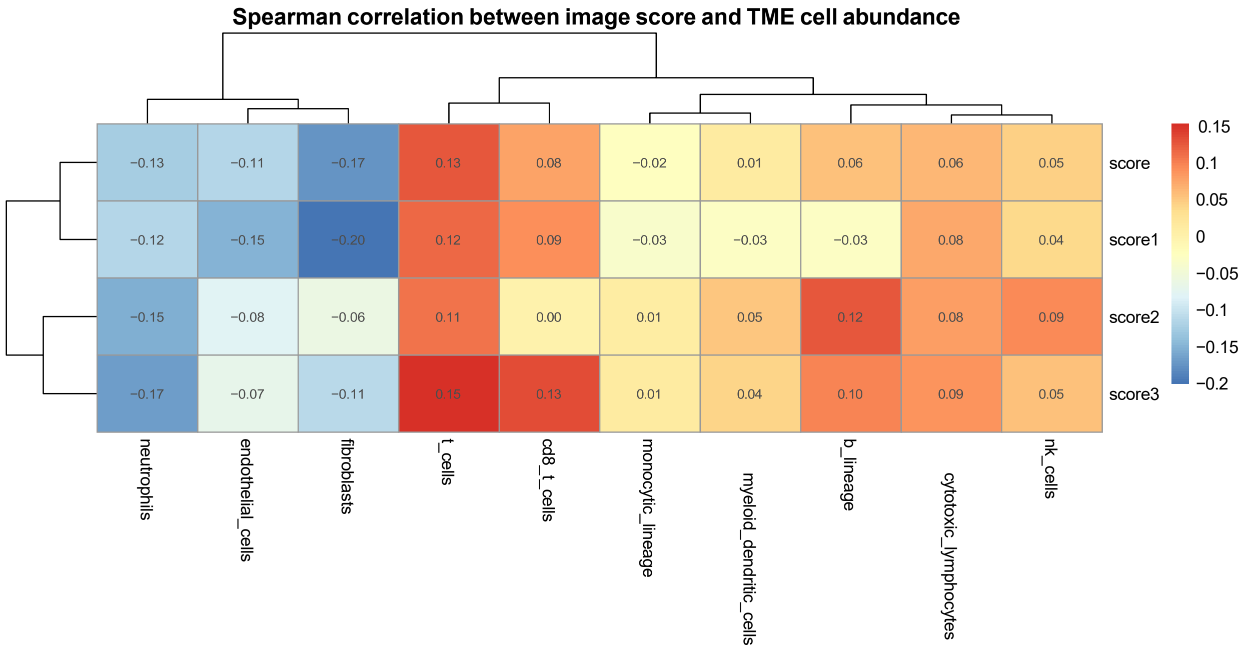

eFigure 23. Correlation Between Tertiary Lymphoid Structure Scores and 10 Tumor Microenvironmental Cell Types Estimated From Gene Expression Data in The Cancer Genome Atlas Stomach Adenocarcinoma Cohort

© 2023 Li Z et al. *JAMA Network Open*.

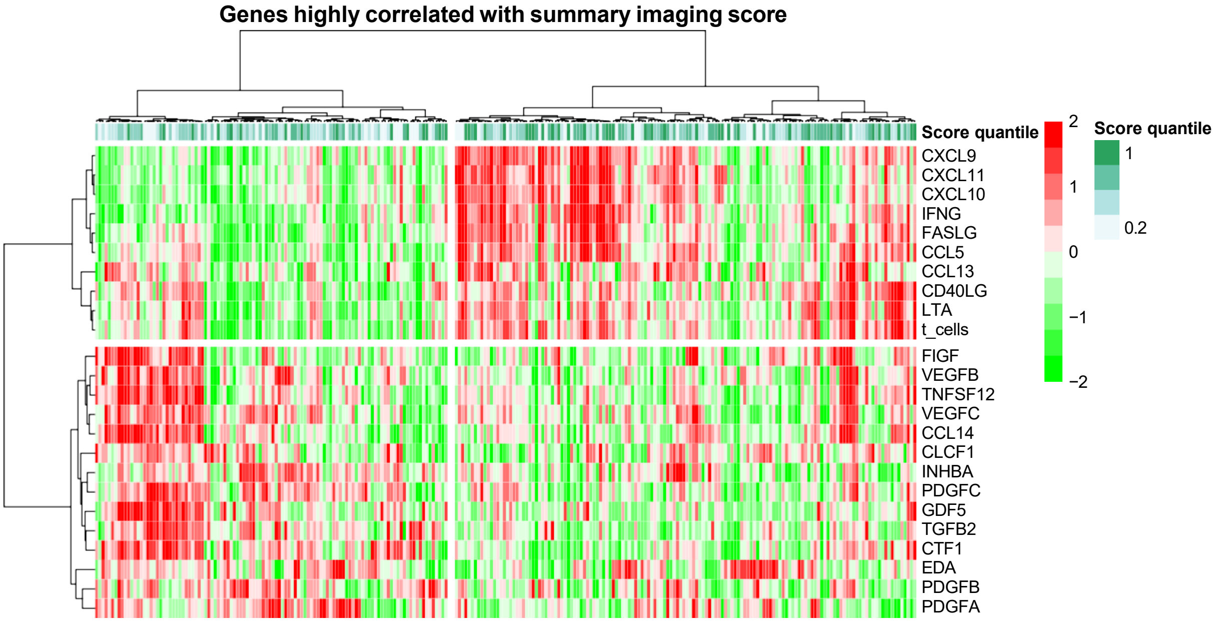

eFigure 24. Gene Expression Profile of 23 Cytokines Correlated With Tertiary Lymphoid Structure Score in The Cancer Genome Atlas Stomach Adenocarcinoma Cohort

© 2023 Li Z et al. *JAMA Network Open*.

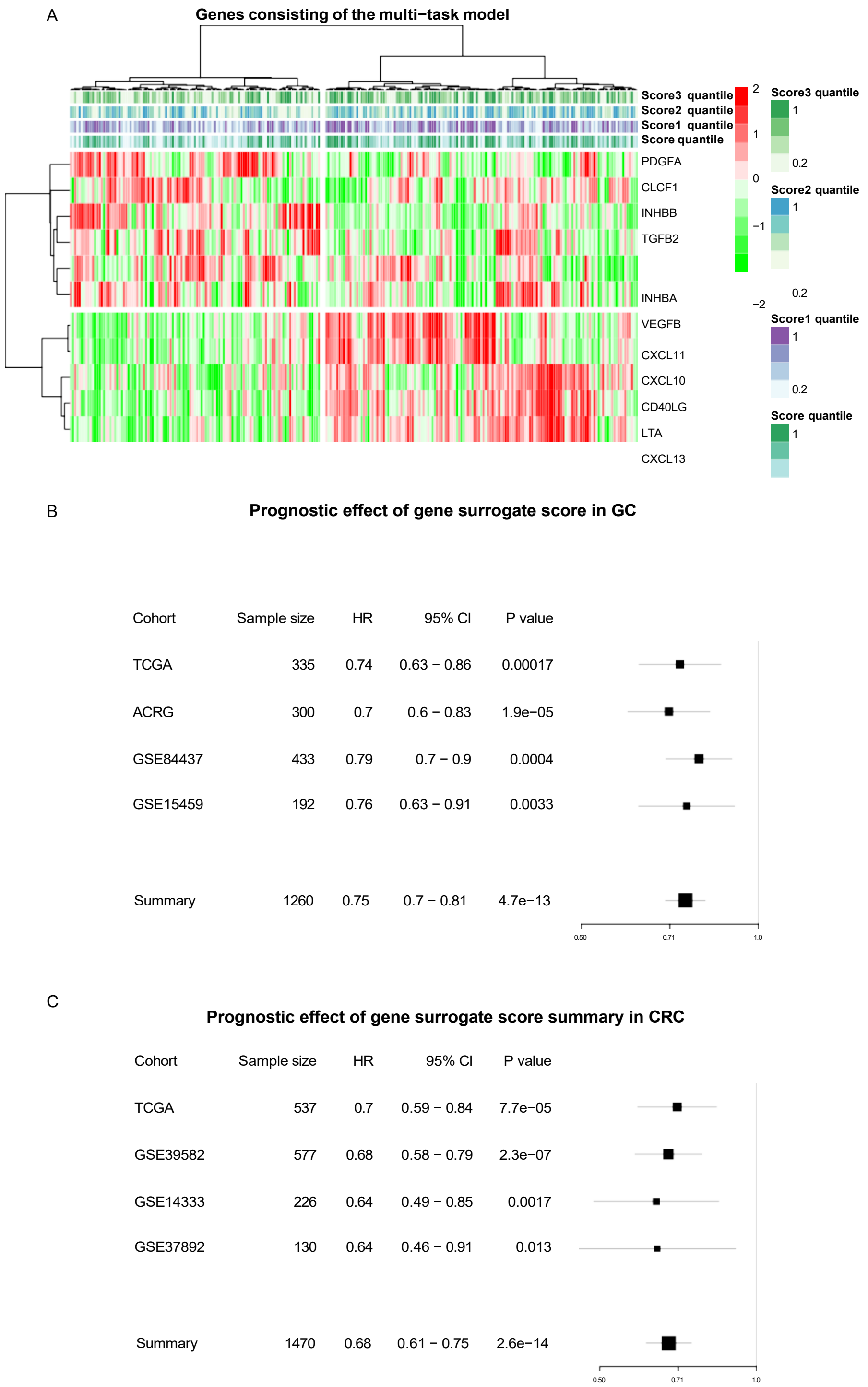

eFigure 25. Molecular Signature of Tertiary Lymphoid Structure Score and Prognostic Value
The distribution of TLS scores and gene expression profile of 11 cytokine genes contained in the TLS signature (A). The prognostic effect of the 11-gene signature in multiple cohorts of gastric (B) and colorectal (C) cancers.

© 2023 Li Z et al. *JAMA Network Open*.

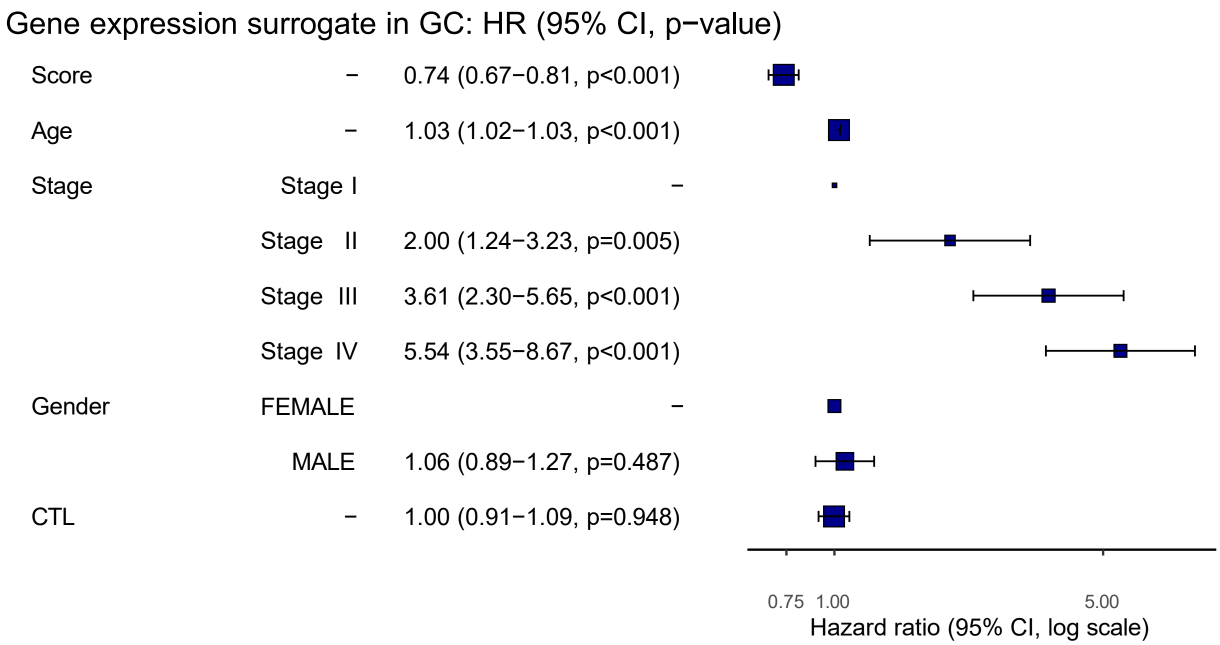

eFigure 26. Multivariate Survival Analysis of Tertiary Lymphoid Structure Molecular Signature in Combined Gastric Cancer Gene Expression Data Sets

CTL: cytotoxic lymphocytes.

© 2023 Li Z et al. *JAMA Network Open*.

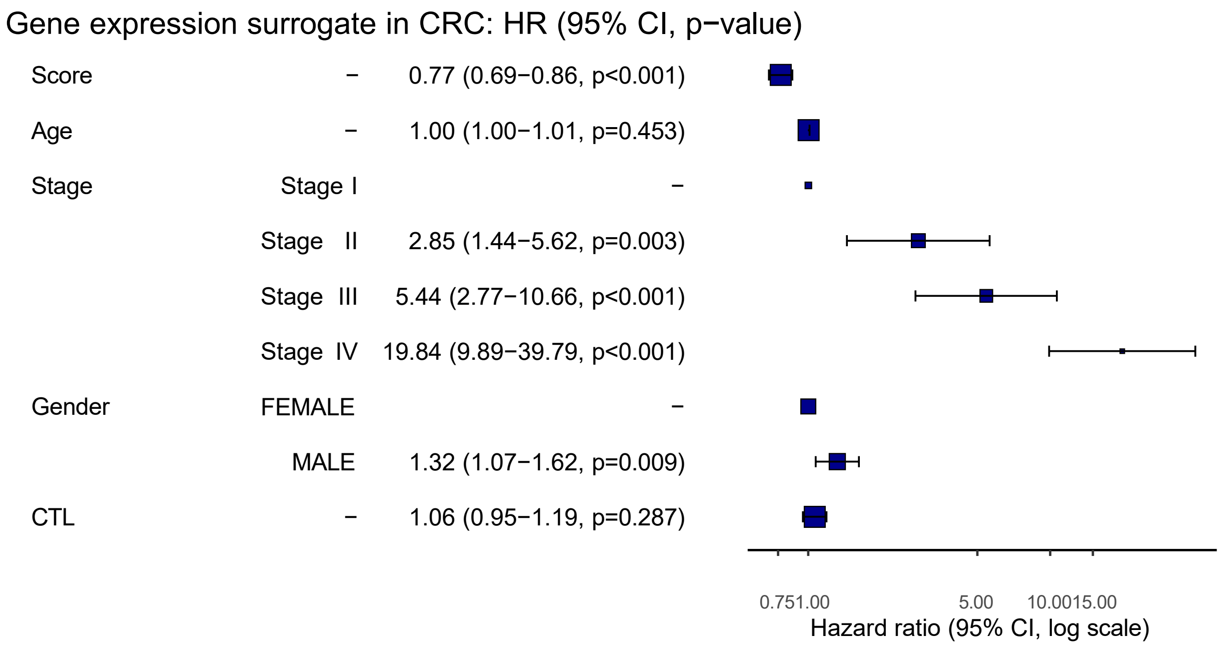

eFigure 27. Multivariate Survival Analysis of Tertiary Lymphoid Structure Molecular Signature in Combined Colorectal Cancer Gene Expression Data Sets

CTL: cytotoxic lymphocytes.

© 2023 Li Z et al. *JAMA Network Open*.

eTables. Supplementary Tables

eTable 1. Association Between Tertiary Lymphoid Structure Score and Tumor Stage or Grade in 7 Cohorts

Variables	TLS score *10^3 (Mean±Std)						
	**TCGA-ESCA**	**TCGA-STAD**	**SMU-STAD**	**TCGA-COAD**	**TCGA-READ**	**TCGA-LIHC**	**TCGA-PAAD**
**Tumor stage**							
Stage I	4.3±6.1	9.7±23.4	12.8±20.6	8.3±16.3	1.1±4.2	2.7±10.4	11.4±22.6
Stage II	3.3±7.6	13.7±27.8	9.4±21.7	6.2±11.3	4.9±11.3	6.1±32.2	9.2±24.7
Stage III	8.6±25.0	13.1±28.3	12.9±25.3	7.2±28.1	6.9±16.2	0.8±2.7	7.8±13.0
Stage IV	4.9±9.0	2.5±4.5	9.6±24.9	6.3±15.4	1.5±2.6	0.8±1.2	1.3±1.8
p	0.187	<0.001	0.540	0.434	0.017	0.190	0.593
**Histological grade**							
G1	3.3±6.3	16.3±31.4	12.3±21.7	-	-	2.2±6.5	7.4±18.3
G2	5.7±20.1	11.5±23.6	11.7±26.8	-	-	3.8±23.3	11.0±29.1
G3	5.6±9.6	12.1±27.8	11.6±23.4	-	-	2.6±11.0	7.2±14.9
G4	-	-	-	-	-	0.4±1.2	0.0±0.0
p	0.084	0.908	0.684	-	-	0.586	0.541

© 2023 Li Z et al. *JAMA Network Open*.

eTable 2. Interslide Variability of Tertiary Lymphoid Structure Scores

	TCGA-LIHC	TCGA-PAAD	TCGA-COAD	TCGA-STAD	TCGA-ESCA	TCGA-READ	SMU-STAD
Average coefficient of variation	0.30	0.40	0.54	0.57	N/A	N/A	N/A
number of patients with >1 slides	7	5	1	20	0	0	0

© 2023 Li Z et al. *JAMA Network Open*.

eTable 3. Univariate and Multivariate Survival Analysis of Individual Tertiary Lymphoid Structure Scores in 7 Cohorts and Combined Data Set

Variables	Univariable			Multivariable		
	HR	95% CI	*p*	HR	95% CI	*p*
**TCGA-ESCA**						
TLS1 score	0.39	0.18,0.84	0.016	0.82	0.46,1.49	0.522
TLS2 score	0.55	0.30,1.01	0.053	0.88	0.55,1.40	0.590
TLS3 score	0.03	0.00,0.49	0.014	0.05	0.00,0.98	0.048
**TCGA-STAD**						
TLS1 score	0.55	0.41,0.75	0.001	0.77	0.56,1.05	0.102
TLS2 score	0.53	0.34,0.82	0.005	0.74	0.50,1.11	0.146
TLS3 score	0.18	0.07,0.51	0.001	0.32	0.12,0.87	0.026
**SMU-STAD**						
TLS1 score	0.68	0.52,0.87	0.003	0.82	0.64,1.05	0.119
TLS2 score	0.54	0.33,0.90	0.017	0.74	0.47,1.17	0.203
TLS3 score	0.15	0.06,0.43	<0.001	0.20	0.07,0.56	0.002
**TCGA-COAD**						
TLS1 score	0.25	0.12,0.52	<0.001	0.33	0.15,0.72	0.005
TLS2 score	0.45	0.18,1.16	0.099	0.89	0.50,1.59	0.703
TLS3 score	0.14	0.03,0.65	0.012	0.45	0.11,1.77	0.252
**TCGA-READ**						
TLS1 score	0.84	0.42,1.67	0.623	1.24	0.87,1.77	0.232
TLS2 score	0.07	0.00, 2.62	0.147	0.07	0.00,2.27	0.132
TLS3 score	0.00	0.00,47.41	0.171	0.00	0.00,45.82	0.181
**TCGA-LIHC**						
TLS1 score	0.18	0.05,0.59	0.005	0.59	0.16,2.14	0.421
TLS2 score	0.09	0.02,0.39	0.001	0.18	0.04,0.89	0.035
TLS3 score	0.00	0.00,0.18	0.019	0.01	0.00,87.93	0.290
**TCGA-PAAD**						
TLS1 score	0.37	0.20,0.69	0.001	0.78	0.45,1.37	0.392
TLS2 score	0.02	0.00,1.04	0.052	0.30	0.03,3.43	0.335
TLS3 score	0.23	0.08,0.63	0.004	0.37	0.13,1.10	0.073
**Meta-analysis**						
TLS1 score	0.61	0.52,0.70	<0.001	0.81	0.71,0.93	0.003
TLS2 score	0.38	0.26,0.55	<0.001	0.66	0.47,0.91	0.011
TLS3 score	0.16	0.09,0.27	<0.001	0.25	0.15,0.42	<0.001

© 2023 Li Z et al. *JAMA Network Open*.

eTable 4. Univariate and Multivariate Survival Analysis of Overall Tertiary Lymphoid Structure Scores in The Cancer Genome Atlas Esophageal Carcinoma

**TCGA-ESCA**	**Univariable**			**Multivariable**		
	HR	95% CI	*p*	HR	95% CI	*p*
**TLS score**	**0.22**	**0.06,0.82**	**0.025**	**0.27**	**0.07,1.04**	**0.058**
Age	1.01	0.98,1.04	0.407	-	-	-
Sex	4.65	1.12,19.23	0.034	2.31	0.47,11.39	0.303
Stage I	0.25	0.06,1.09	0.065	1		
Stage II	0.46	0.25,0.86	0.014	3.46	0.64,18.71	0.150
Stage III	2.56	1.40,4.68	0.002	9.10	1.64,50.44	0.016
Stage IV	2.98	1.16,7.66	0.023	13.22	2.01,86.84	0.007
G1	0.51	0.18,1.45	0.206	1		
G2	1.22	0.67,2.24	0.512	0.95	0.30,3.01	0.933
G3	1.11	0.58,2.13	0.752	0.84	0.23,3.01	0.785
TIL score	1.27	0.91,1.78	0.161	-	-	-

© 2023 Li Z et al. *JAMA Network Open*.

eTable 5. Univariate and Multivariate Survival Analysis of Overall Tertiary Lymphoid Structure Scores in The Cancer Genome Atlas Stomach Adenocarcinoma

**TCGA-STAD**	**Univariable**			**Multivariable**		
	HR	95% CI	*p*	HR	95% CI	*p*
**TLS score**	**0.28**	**0.15,0.53**	**<0.001**	**0.29**	**0.16,0.55**	**<0.001**
Age	1.02	1.01,1.04	0.006	1.04	1.02,1.05	<0.001
Sex	1.10	0.77,1.57	0.604	-	-	-
Stage I	0.37	0.18,0.76	0.007	1		
Stage II	0.66	0.45,0.99	0.044	2.16	0.98,4.75	0.057
Stage III	1.37	0.98,1.92	0.067	3.35	1.59,7.05	0.001
Stage IV	2.43	1.53,3.88	<0.001	6.68	2.87,15.52	<0.001
G1	0.63	0.16,2.55	0.518	1		
G2	0.79	0.55,1.13	0.198	0.90	0.22,3.75	0.883
G3	1.31	0.91,1.87	0.141	1.21	0.30,4.97	0.788
TIL score	0.90	0.75,1.08	0.240	-	-	-

© 2023 Li Z et al. *JAMA Network Open*.

eTable 6. Univariate and Multivariate Survival Analysis of Overall Tertiary Lymphoid Structure Scores in Southern Medical University Stomach Adenocarcinoma

**SMU-STAD**	**Univariable**			**Multivariable**		
	HR	95% CI	*p*	HR	95% CI	*p*
**TLS score**	**0.30**	**0.18,0.50**	**<0.001**	**0.32**	**0.19,0.53**	**<0.001**
Age	1.00	0.98,1.01	0.644	-	-	-
Sex	0.83	0.58,1.19	0.318	-	-	-
Stage I	0.24	0.11,0.54	<0.001	1		
Stage II	0.71	0.46,1.10	0.125	2.11	0.86,5.19	0.103
Stage III	1.17	0.83,1.64	0.373	3.39	1.46,7.84	0.004
Stage IV	3.24	2.14,4.90	<0.001	8.32	3.42,20.24	<0.001
G1	0.33	0.15,0.71	0.004	1		
G2	0.86	0.56,1.32	0.494	2.11	0.91,4.86	0.081
G3	1.74	1.17,2.57	0.006	2.71	1.25,5.86	0.011
TIL score	0.98	0.83,1.16	0.816	-	-	-

© 2023 Li Z et al. *JAMA Network Open*.

eTable 7. Univariate and Multivariate Survival Analysis of Overall Tertiary Lymphoid Structure Scores in The Cancer Genome Atlas Colon Adenocarcinoma

**TCGA-COAD**	**Univariable**			**Multivariable**		
	HR	95% CI	*p*	HR	95% CI	*p*
**TLS score**	**0.07**	**0.02,0.33**	**<0.001**	**0.11**	**0.02,0.47**	**0.003**
Age	1.02	1.00,1.04	0.018	1.03	1.01,1.05	0.001
Sex	1.18	0.77,1.81	0.459	-	-	-
Stage I	0.27	0.10,0.74	0.011	1		
Stage II	0.48	0.30,0.78	0.003	1.68	0.58,4.93	0.342
Stage III	1.21	0.77,1.89	0.407	3.64	1.27,10.42	0.016
Stage IV	4.09	2.60,6.44	<0.001	9.46	3.31,27.05	<0.001
TIL score	0.86	0.67,1.10	0.226	-	-	-

© 2023 Li Z et al. *JAMA Network Open*.

eTable 8. Univariate and Multivariate Survival Analysis of Overall Tertiary Lymphoid Structure Scores in The Cancer Genome Atlas Rectum Adenocarcinoma

**TCGA-READ**	**Univariable**			**Multivariable**		
	HR	95% CI	*p*	HR	95% CI	*p*
**TLS score**	**0.01**	**0.00,0.68**	**0.033**	**0.01**	**0.00,1.42**	**0.067**
Age	1.08	1.03,1.14	0.004	1.06	1.01,1.12	0.028
Sex	0.88	0.39,2.00	0.757	-	-	-
Stage I	0.47	0.11,2.00	0.305	1		
Stage II	0.38	0.13,1.12	0.078	1.09	0.20,6.02	0.923
Stage III	0.86	0.35,2.11	0.747	1.85	0.37,9.29	0.455
Stage IV	4.92	2.11,11.47	<0.001	5.63	1.21,26.16	0.027
TIL score	0.66	0.37,1.18	0.158	-	-	-

© 2023 Li Z et al. *JAMA Network Open*.

eTable 9. Univariate and Multivariate Survival Analysis of Overall Tertiary Lymphoid Structure Scores in The Cancer Genome Atlas Liver Hepatocellular Carcinoma

**TCGA-LIHC**	**Univariable**			**Multivariable**		
	HR	95% CI	*p*	HR	95% CI	*p*
**TLS score**	**0.00**	**0.00,0.16**	**0.006**	**0.00**	**0.00,0.22**	**0.008**
Age	1.01	1.00,1.03	0.155	-	-	-
Sex	0.75	0.51,1.09	0.133	-	-	-
Stage I	0.47	0.32,0.70	<0.001	1		
Stage II	0.95	0.61,1.49	0.835	1.65	0.99,2.75	0.055
Stage III	2.28	1.56,3.33	<0.001	2.67	1.71,4.16	<0.001
Stage IV	3.82	1.21,12.09	0.023	5.27	1.59,17.47	0.007
G1	0.92	0.51,1.64	0.767	1		
G2	0.94	0.65,1.36	0.747	1.13	0.61,2.10	0.688
G3	1.06	0.72,1.56	0.763	1.30	0.69,2.45	0.416
G4	1.34	0.54,3.30	0.524	2.07	0.71,6.04	0.181
TIL score	0.86	0.68,1.08	0.196	-	-	-

© 2023 Li Z et al. *JAMA Network Open*.

eTable 10. Univariate and Multivariate Survival Analysis of Overall Tertiary Lymphoid Structure Scores in The Cancer Genome Atlas Pancreatic Adenocarcinoma

TCGA-PAAD	Univariable			Multivariable		
	HR	95% CI	*p*	HR	95% CI	*p*
**TLS score**	**0.11**	**0.03,0.45**	**0.002**	**0.12**	**0.03,0.47**	**0.002**
Age	1.02	1.00,1.04	0.030	1.01	0.99,1.03	0.355
Sex	0.91	0.61,1.38	0.667	-	-	-
Stage I	0.30	0.12,0.75	0.010	1		
Stage II	2.44	1.22,4.87	0.012	2.79	1.11,7.04	0.029
Stage III	0.51	0.07,3.66	0.503	1.19	0.14,10.41	0.878
Stage IV	1.09	0.34,3.46	0.883	2.42	0.57,10.31	0.232
G1	0.55	0.29,1.03	0.062	1		
G2	0.97	0.64,1.46	0.873	1.55	0.79,3.01	0.200
G3	1.52	0.99,2.34	0.055	2.29	1.12,4.65	0.022
G4	0.83	0.11,5.95	0.850	0.87	0.11,6.84	0.896
TIL score	1.19	0.97,1.46	0.099	-	-	-

© 2023 Li Z et al. *JAMA Network Open*.

eTable 11. The 11 Cytokine Genes and Corresponding Weights in Tertiary Lymphoid Structure Molecular Signature

Gene	Score1	Score2	Score3	Overall Score
INHBB	-0.0011925	-0.0213586	0.05558115	0.03667404
INHBA	-0.0234867	-0.0066676	-0.0287959	-0.0534208
TGFB2	-0.0493959	-0.0130274	-0.0588854	-0.1098391
CD40LG	0.00999738	0.01121993	0.02234881	0.03987143
LTA	0.00700517	0.01178709	0.01304928	0.02862462
VEGFB	-0.051586	-0.0365246	-0.1074193	-0.1798846
PDGFA	-0.0029008	0.00519719	-0.0171173	-0.0151014
CLCF1	-0.0305246	0.00729532	-0.0220226	-0.0406194
CXCL13	0.00101962	0.02839857	0.03230528	0.05698597
CXCL11	0.00173586	-0.0017343	0.00775286	0.00770211
CXCL10	0.04781635	-0.0260708	0.09173395	0.10856572

© 2023 Li Z et al. *JAMA Network Open*.
