# Supplementary material for: Development and Validation of a Machine Learning Model for Detection and Classification of Tertiary Lymphoid Structures in Gastrointestinal Cancers
Source: JAMA Netw Open. 2023 Jan 24;6(1):e2252553. doi: 10.1001/jamanetworkopen.2022.52553 (PMC10408275; doi:10.1001/jamanetworkopen.2022.52553)
Supplement: Supplement 2. — Data Sharing Statement [file jamanetwopen-e2252553-s002.pdf]

Data Sharing Statement

Li. Development and Validation of a Machine Learning Model for Detection and Classification of Tertiary Lymphoid Structures in Gastrointestinal Cancers. Published January 24, 2023.
doi:10.1001/jamanetworkopen.2022.52553

Data
Data available: Yes
Data types: Deidentified participant data, Data dictionary **How to access data:** rli2@stanford.edu **When available:** With publication

Supporting Documents
Document types: None

Additional Information
Who can access the data: researchers whose proposed use of the data has been approved
Types of analyses: for any purpose
Mechanisms of data availability: with a signed data access agreement

© 2023 Li Z et al. *JAMA Network Open*.
